# Ulvan Microparticles for Encapsulation of Eucalyptus Essential Oil: Characterization, Release Behavior, and Stability in Aquafeeds

**DOI:** 10.1155/anu/3902818

**Published:** 2026-08-09

**Authors:** Nikoletta Tricha, Chrysanthos Stergiopoulos, Sofia Papadaki, Apostolos Tsoumanis, Magdalini Krokida

**Affiliations:** ^1^ Laboratory of Process Analysis and Design, School of Chemical Engineering, National Technical University of Athens, Iroon Polytechneiou 9, Athens 157 80, Greece, ntua.gr; ^2^ DIGNITY Private Company, 30–32 Leoforos Alexandrou Papagou, Zografou, Athens 157 71, Greece

**Keywords:** controlled delivery systems, functional feeds, oxidative stability, phytobiotics

## Abstract

Ulvan–lecithin microstructures were developed as a delivery platform for eucalyptus essential oil (EEO) in functional aquafeeds to improve phytobiotic retention, modulate gastrointestinal release, and enhance pellet stability during storage. Preliminary formulation screening compared ulvan‐only and ulvan–lecithin systems containing different lecithin levels. Among the tested formulations, ulvan–1%–lecithin–EEO (U–L1–EEO) showed the most favorable overall profile, with the highest encapsulation efficiency (EE%, 76.4% ± 1.2%), the lowest surface‐associated EEO fraction (23.6% ± 1.2%), a median particle size of 145.2 ± 4.8 μm, a narrow size distribution (span = 1.33 ± 0.04), and a negative ζ‐potential (−31.6 ± 1.8 mV). Fourier‐transform infrared (FTIR) spectroscopy and differential scanning calorimetry (DSC) analyses supported the formation of a physically stabilized ulvan–lecithin matrix and the incorporation of EEO through mainly noncovalent interactions. In PBS (pH 7.4), U–L1–EEO exhibited sustained release, reaching ~90% after 24 h, with Higuchi and Korsmeyer–Peppas modeling indicating diffusion‐governed and anomalous transport behavior during the initial release phase. Under simulated *Dicentrarchus labrax* gastrointestinal conditions, release was limited during the gastric phase (~12.7% at 90 min, pH 2.5) and increased markedly under intestinal conditions (~82% at 360 min, pH 7.5 with pancreatin and bile salts), indicating preferential intestinal release under the applied in vitro conditions. When incorporated into commercial aquafeed pellets at equivalent EEO inclusion (0.1% w/w), the encapsulated formulation improved accelerated storage stability (45°C, 70% RH, 28 days), reducing moisture uptake and peroxide value (PV) development while improving lipid and ω−3 retention, antioxidant capacity, total phenolic content (TPC), pellet durability, hardness, fines resistance, and water stability. Ulvan–lecithin microencapsulation appears to be a promising proof‐of‐concept strategy for stabilizing volatile phytobiotics and improving the storage performance of functional aquafeeds, although in vivo validation and process‐scale assessment are required before biological efficacy and industrial applicability can be confirmed.

## 1. Introduction

Aquaculture has become the fastest‐growing animal protein sector globally, currently accounting for more than half of aquatic food consumed worldwide [1]. In Mediterranean production systems, species such as European sea bass (*Dicentrarchus labrax*) and gilthead sea bream (*Sparus aurata*) dominate the output. However, intensification of farming practices has been accompanied by increased disease susceptibility, oxidative stress, and reduced physiological resilience, necessitating functional feed strategies that enhance fish health and robustness [2].

Historically, disease management relied heavily on antibiotics, but concerns regarding antimicrobial resistance, environmental impact, and regulatory restrictions within the European Union have accelerated the search for sustainable alternatives [3]. Phytobiotics have emerged as promising candidates due to their antimicrobial, antioxidant, and immunomodulatory activities. Eucalyptus essential oil (EEO, *Eucalyptus globulus*) and bioactive compounds derived from the green macroalga *Ulva* spp. exhibit documented biological effects relevant to aquaculture applications [4, 5]. Nevertheless, the practical incorporation of phytobiotics into aquafeeds remains technologically challenging. Volatile essential oil constituents are highly sensitive to oxygen, light, and heat, leading to substantial losses during processing and storage [6], while their hydrophobic nature limits dispersion and bioavailability within feed matrices.

Encapsulation offers a viable strategy to address these limitations by protecting sensitive compounds and enabling controlled release. Natural marine biopolymers are particularly attractive as encapsulation matrices due to their biodegradability, biocompatibility, and functional bioactivity. Seaweed‐derived ingredients are increasingly being investigated for aquafeed applications due to their nutritional value, functional polysaccharides, antioxidant compounds, and potential prebiotic or immunomodulatory effects [7]. Green macroalgae have also been evaluated as alternative feed ingredients, with previous studies reporting their proximate composition and amino acid quality for aquafeed use [8]. Among seaweed‐derived compounds, ulvan, a sulfated polysaccharide extracted from *Ulva lactuca*, possesses intrinsic antioxidant and immunomodulatory properties and exhibits structural characteristics suitable for hydrogel formation and matrix stabilization [9, 10]. Ulvan‐derived compounds have also shown immunostimulatory potential in aquatic species, supporting their relevance for functional feed applications [11]. Despite its promising attributes, ulvan remains underexplored as a carrier for volatile phytobiotics in aquafeed systems.

To date, limited studies have investigated the combined evaluation of encapsulation efficiency (EE%), gastrointestinal release behavior, oxidative stability, and pellet technological integrity within real aquafeed formulations. In particular, combined ulvan–lecithin matrices for encapsulating essential oils have not been systematically investigated in aquafeed systems. Lecithin may support interfacial stabilization of hydrophobic essential oil droplets, whereas ulvan may provide a marine‐derived polysaccharide network with potential bioactivity and matrix‐forming capacity [12–14]. Existing studies have mainly addressed seaweed‐derived ingredients, ulvan bioactivity, or essential oil encapsulation separately, while limited information is available on integrated evaluation of gastrointestinal release, retention of bioactive compounds, preservation of nutritionally critical polyunsaturated fatty acids, antioxidant functionality, oxidative stability, and pellet technological integrity during storage [7, 15–17]. To the best of our knowledge, studies that combine ulvan–lecithin essential oil encapsulation with a *Dicentrarchus labrax*‐relevant gastrointestinal model and accelerated aging of real commercial pellets, while simultaneously assessing oxidative, nutritional, antioxidant, and mechanical integrity metrics, remain scarce.

Therefore, the present study aims to develop ulvan–lecithin microparticles for the encapsulation of EEO and to evaluate their performance within commercial aquafeed matrices. Specifically, we assess (i) physicochemical and thermal characteristics of the microstructures, (ii) release kinetics under simulated aqueous and gastrointestinal conditions relevant to *Dicentrarchus labrax*, and (iii) oxidative, nutritional, and technological stability of supplemented feeds under accelerated aging. This integrated evaluation seeks to provide a proof‐of‐concept assessment of a marine‐derived encapsulation platform to enhance phytobiotic delivery and storage stability in Mediterranean aquaculture systems.

## 2. Materials and Methods

### 2.1. Characterization of EEO

GC–MS analysis for the determination of the compound profile of the EEO was performed by using a GC‐2030 gas chromatographic system coupled to a GCMS‐QP2020 NX single quadrupole mass detector (Shimadzu Corporation, Kyoto, Japan), equipped with an SP‐2340 30 m × 20 μm × 0.25 μm fused silica capillary column (Supelco, Bellefonte, PA, US). The carrier gas was helium (He), with a flow rate of 1 mL/min in the splitless mode. The temperature of the column started at 43°C and rose without a hold time from 43 to 60°C at 2.5°C/min, then to 230°C at 15°C/min. The temperature was then maintained at a constant value for 12 min. Subsequently, the oven temperature was raised to 240°C for a post‐run period of 5 min for column clean‐up. Quantification was performed by external calibration using analytical standards over the concentration range of 10–500 mg L^−1^, yielding a linear calibration curve with *R* ^2^ > 0.99.

### 2.2. Preparation and Preliminary Screening of Phytobiotic Microstructures

EEO (*Eucalyptus globulus*, food‐grade, Syndesmos S.A., Greece) was encapsulated within ulvan‐based polysaccharide matrices using an emulsion‐assisted freeze‐drying method. A preliminary formulation screening was first conducted to evaluate the effect of lecithin incorporation on the basic properties of the microstructures. Four formulations were prepared: ulvan–EEO without lecithin (U–EEO), ulvan–0.5% lecithin–EEO (U–L0.5–EEO), ulvan–1% lecithin–EEO (U–L1–EEO), and ulvan–2% lecithin–EEO (U–L2–EEO). In all formulations, ulvan, previously extracted from *Ulva lactuca*, was dissolved in deionized water at 5% (w/v) and continuously stirred at 45°C until complete solubilization. Soy lecithin was incorporated at 0, 0.5, 1.0, or 2.0% (w/w relative to ulvan) and dispersed under stirring prior to oil addition. EEO was gradually added at 10% (w/w relative to ulvan), corresponding to a polymer‐to‐oil mass ratio of 10:1, while the aqueous phase was maintained under agitation to promote pre‐emulsification. The mixture was homogenized using an Ultra‐Turrax T25 homogenizer (IKA, Germany) at 15,000 rpm for 10 min to form an oil‐in‐water emulsion. Subsequently, an ultrasonic bath treatment was applied for 5 min at 50 kHz to reduce the droplet size and enhance dispersion stability. The resulting emulsions were poured into stainless‐steel trays, frozen at −80°C for 24 h, and freeze‐dried using a laboratory freeze‐dryer (Bk‐Fd10s, BIOBASE, China) for 48 h at a chamber pressure below 0.1 mbar and a condenser temperature of −50°C. No programed shelf‐temperature ramp was applied; the process was performed under the available fixed laboratory freeze‐dryer operating conditions. The obtained dry porous matrices were gently milled to yield free‐flowing microparticle powders, which were stored in airtight containers at 4°C until further analysis. All formulations were prepared in triplicate to ensure reproducibility. Based on the preliminary screening of EE%, surface oil content, particle size distribution, and ζ‐potential, the U–L1–EEO formulation was selected for subsequent detailed physicochemical characterization, in vitro release testing, simulated gastrointestinal digestion, and aquafeed incorporation.

### 2.3. Characterization of Screening Formulations and Selected Phytobiotic Microstructure

#### 2.3.1. Particle Size Distribution

Particle size distribution of the freeze‐dried microencapsulated powders was determined for all preliminary screened formulations by laser diffraction using a Microtrac S3500 analyzer (Microtrac Retsch GmbH, Haan, Germany) equipped with a wet dispersion module. Samples were dispersed in deionized water with moderate stirring and brief sonication (5 min) to ensure adequate deagglomeration without inducing particle fragmentation, simulating hydrated conditions relevant to the release behavior. The concentration was adjusted to achieve an optimal obscuration range (10%–15%) prior to the measurement. The median particle diameter (*d*_50_), as well as the *d*_10_ and *d*_90_ percentiles, was recorded. Particle size distribution width was expressed as a span, calculated as:

(1) Span=d90−d10d50.

All measurements were conducted in triplicate, and results are reported as the mean ± standard deviation.

#### 2.3.2. Zeta Potential

The zeta potential of all preliminary screening formulations was determined using a Zetasizer Nano ZS (Malvern Instruments, UK) based on electrophoretic light scattering. Prior to analysis, samples were dispersed in deionized water at a 1:10 dilution and gently vortexed to ensure uniform particle dispersion. Measurements were performed at 25°C using a folded capillary cell, and results were expressed as the mean ± standard deviation of three independent measurements. The values reflect the surface charge of dispersed fragments.

#### 2.3.3. Surface Oil and EE%

EE% and surface oil content were determined for all screened formulations by quantifying the surface‐associated, non‐encapsulated fraction of EEO from the freeze‐dried microcapsules. Approximately 50 mg of powder was extracted with 5 mL of n‐hexane, vortex‐mixed for 1 min, and ultrasonicated for 10 min to recover the readily extractable oil located on or near the particle surface. Samples were centrifuged at 5000 rpm for 10 min, and the supernatant was filtered through a 0.22 µm PTFE membrane prior to analysis. The EEO concentration was quantified by gas chromatography–mass spectrometry (GC–MS; Shimadzu GC‐2030 coupled with a QP2020 NX mass detector) using helium as the carrier gas with splitless injection. Quantification was performed by external calibration using eucalyptol standards. The total oil content was determined based on the amount of EEO incorporated during microstructure preparation. Surface oil content and EE% were calculated as follows:

(2) Surface Oil %=Surface OilTotal Oil ⋅100

(3) EE %=Total oil−Surface oilTotal oil⋅100 .

All measurements were performed in triplicate, and results are reported as the mean ± standard deviation.

#### 2.3.4. Fourier‐Transform Infrared (FTIR) Spectroscopy

FTIR spectra were recorded using a JASCO FT/IR‐4200 spectrometer (JASCO Corp., Japan). Spectra were collected for ulvan, lecithin, pure EEO, the unloaded ulvan–lecithin matrix, and the selected U–L1–EEO microencapsulated formulation. Samples were prepared as KBr pellets by mixing the dried powder with spectroscopic‐grade KBr at approximately a 1:100 (w/w) ratio and compressing under hydraulic pressure to obtain transparent discs. Pure EEO was analyzed separately using ATR‐FTIR, with the reference material supporting the interpretation of oil‐related absorption bands in the final encapsulated system. Spectra were collected over the range of 4000–400 cm^−1^ at a spectral resolution of 4 cm^−1^. The background spectra were recorded prior to each measurement and automatically subtracted. FTIR analysis was performed to identify characteristic functional groups and to evaluate potential intermolecular interactions among ulvan, lecithin, and EEO within the microencapsulated structures.

#### 2.3.5. Differential Scanning Calorimetry (DSC)

Thermal properties of the samples were analyzed using a DSC (PerkinElmer DSC 6) under a nitrogen atmosphere (20 mL/min). DSC analysis was performed for ulvan, pure EEO, the unloaded ulvan–lecithin matrix, and the selected U–L1–EEO microencapsulated formulation. Approximately 5–8 mg of the sample was sealed in aluminum DSC pans. Samples were equilibrated at −10°C for 1 min and then heated to 250°C at 5°C/min. After cooling to −10°C, a second heating cycle was performed up to 350°C under the same conditions. Thermal transitions observed during the heating cycles were recorded and used to evaluate the changes in the thermal behavior of the microencapsulated systems. Pure EEO was included as a reference material because it is a volatile liquid mixture rather than a polymeric matrix; therefore, its thermal profile was interpreted primarily in terms of volatilization‐associated events rather than glass‐transition behavior.

### 2.4. In Vitro Release Study

The release behavior of EEO from the selected U–L1–EEO microencapsulated formulation was evaluated under controlled conditions in phosphate‐buffered saline (PBS, pH 7.4) supplemented with 0.25% (v/v) Tween 80 to enhance solubilization and maintain sink conditions of the lipophilic compounds. U–L1–EEO (100 mg) was dispersed in 50 mL of release medium and incubated at 25 ± 1°C under constant agitation (100 rpm) to approximate typical seawater temperatures encountered in Mediterranean aquaculture systems, where European sea bass (*Dicentrarchus labrax*) is commonly cultured, with optimal growth and metabolic activity generally observed between ~22 and 25°C [18]. At predetermined time intervals (15–1440 min), 1 mL aliquots were withdrawn and immediately replaced with an equal volume of fresh medium to maintain a constant volume. Cumulative release values were corrected for dilution effects resulting from sample withdrawal. Samples were centrifuged, and the supernatant was analyzed by GC–MS to quantify the EEO. Cumulative release (%) was calculated relative to the experimentally determined total encapsulated EEO content. Release kinetics were analyzed using zero‐order, first‐order, Higuchi, and Korsmeyer–Peppas models. Zero‐order and first‐order models were used as empirical reference models for constant‐rate and concentration‐dependent release, respectively, whereas the Higuchi and Korsmeyer–Peppas models were selected to assess diffusion‐controlled and anomalous transport from the hydrated ulvan–lecithin matrix [19–21]. Nonlinear regression analysis was performed, and model performance was evaluated based on the coefficient of determination (*R* ^2^) and the root mean square error (RMSE). The Korsmeyer–Peppas model was applied exclusively to the initial release stage (*R*_t_ < 60%), in accordance with its theoretical validity range for diffusion‐controlled systems. The complete release dataset used for kinetic modeling is provided in Table S1.

### 2.5. In Vitro Gastrointestinal Release Simulation

An in vitro gastrointestinal release study was conducted to simulate the digestive conditions of *Dicentrarchus labrax* using a two‐stage sequential protocol comprising the gastric and intestinal phases. For the gastric phase, U–L1–EEO powder (100 mg) was suspended in 50 mL of simulated gastric fluid (pH 2.5) containing NaCl (0.2% w/v) and pepsin (activity 0.8 FIP U mg^−1^; Sigma–Aldrich, St. Louis, MO, USA) at a final concentration of 2 mg mL^−1^. The suspension was incubated at 25 ± 1°C under gentle agitation (100 rpm) for 90 min. The selected pH and residence time were chosen to approximate reported gastric conditions and transit durations for marine carnivorous fish under feeding conditions. The selected temperature was chosen to approximate physiological conditions for *D. labrax*, as fish are ectothermic organisms and their internal temperature closely follows the surrounding water temperature, which in Mediterranean aquaculture typically ranges between 22 and 25°C [18]. Following completion of the 90 min gastric stage, the medium pH was adjusted to 7.5 using NaOH. Pancreatin (activity 54.6 FIP U mg^−1^; ITW Reagents, Barcelona, Spain) at a final concentration of 2 mg mL^−1^, together with bile salts (5 mM), was then added to simulate intestinal conditions. The intestinal phase was continued until a total digestion time of 360 min under identical agitation conditions. At predetermined intervals during both phases, 1 mL aliquots were withdrawn and immediately replaced with an equal volume of fresh medium to maintain constant volume. Cumulative release values were corrected for dilution effects caused by repeated sampling. Samples were centrifuged, and the supernatant was analyzed by GC–MS to quantify the released EEO. The release data were analyzed separately for the gastric and intestinal phases using the same kinetic modeling approach described for the PBS release study. The complete gastrointestinal release dataset used for kinetic modeling is provided in Table S2.

### 2.6. Preparation and Stability Assessment of Functional Aquafeeds

#### 2.6.1. Feed Formulation

The selected U–L1–EEO microencapsulated formulation was incorporated into commercial aquafeed pellets (1.7 mm; Zoonomi S.A.). Three experimental diets were prepared: a control diet without phytobiotic supplementation, a diet supplemented with free EEO, and a diet supplemented with encapsulated EEO. The ingredient composition is presented in Table 1. Formulations were designed to be isonitrogenous and isolipidic, with the total composition being maintained at 100% (w/w) in all cases. Phytobiotic inclusion partially replaced wheat flour in the supplemented diets to maintain the mass balance.

**Table 1:** Ingredient composition of the experimental diets (% w/w).

Ingredient	Functional feed with free EEO (%)	Functional feed with encapsulated EEO (%)	Control (%)
Fish meal	50.0	50.0	50.0
Fish oil	10.0	10.0	10.0
Corn gluten meal	8.0	8.0	8.0
Wheat gluten flour	5.0	5.0	5.0
Concentrated soy protein	6.0	6.0	6.0
High protein sunflower flour	5.0	5.0	5.0
Wheat flour	12.90	11.50	13.0
Vitamins and minerals premix	3.0	3.0	3.0
Phytobiotics	0.1	1.5	0.0

The phytobiotic inclusion level was set at 0.1% (w/w) for both the free and encapsulated EEO diets, corresponding to an equivalent EEO content. The amount of encapsulated powder was adjusted accordingly based on the EE% of the selected U–L1–EEO formulation. This inclusion level was selected based on literature‐reported effective doses for phytobiotic applications in aquafeeds and preliminary stability considerations. The phytobiotic additives were incorporated into the feed formulation during the mixing stage prior to extrusion. Encapsulated EEO was added in powder form as the selected U–L1–EEO microencapsulated formulation, whereas free EEO was introduced directly as a liquid additive to support its distribution within the feed mixture. The resulting mixtures were subsequently processed by extrusion under standard aquafeed extrusion conditions, with a conditioning temperature of ~120°C [22]. This approach enabled the evaluation of the physicochemical, oxidative, antioxidant, nutritional, and technological performance of the final pellets. However, direct GC–MS quantification of EEO retention immediately after extrusion was not performed; therefore, retention during extrusion and the complete structural preservation of the encapsulated system during processing cannot be conclusively confirmed.

#### 2.6.2. Accelerated Aging Conditions

Pellets were subjected to accelerated aging in a controlled‐environment climatic chamber (45 ± 1°C and 70 ± 2% relative humidity) for 28 days. These conditions were selected to simulate long‐term storage stress and accelerate moisture uptake and oxidative degradation. For each formulation, ~200 g of pellets were evenly distributed in perforated stainless‐steel trays to allow uniform air circulation and exposure to controlled humidity. Samples were stored without packaging to simulate the worst‐case environmental exposure. At predetermined intervals (0, 3, 7, 10, 14, and 28 days), independent subsamples were withdrawn (destructive sampling) and immediately sealed in airtight containers prior to analysis. Samples were equilibrated to room temperature before physicochemical and oxidative measurements. All the analytical determinations described below were performed in triplicate, unless otherwise stated.

### 2.7. Pellet Functional Stability

#### 2.7.1. Moisture Content

The moisture content was determined using a halogen moisture analyzer (KERN DBS 60‐3). Approximately 0.50 ± 0.01 g of the ground pellet sample was heated at 102°C until constant mass (mass variation < 1 mg over 30 s). The results were expressed as % (w/w).

#### 2.7.2. Crude Protein

Crude protein was quantified by Kjeldahl nitrogen determination according to the AOAC Official Method 981.10 [23]. Samples were digested with H_2_SO_4_ in the presence of K_2_SO_4_ and CuSO_4_ catalysts, then steam‐distilled into standard H_2_SO_4_ and titrated with 0.5 N NaOH using a methyl‐red/methylene‐blue mixed indicator. The nitrogen content was converted to protein using a conversion factor of 6.25. The analyses were conducted in triplicate.

#### 2.7.3. Crude Lipid Content and Fatty Acid Profile

Total lipids were extracted from ground pellets using a modified Bligh and Dyer method [24] (chloroform:methanol:water, 2:1:0.8 v/v/v). The extracted lipids were evaporated under nitrogen and weighed gravimetrically. Fatty acid methyl esters (FAMEs) were prepared by base‐catalyzed transmethylation using methanolic KOH and analyzed by GC–MS (Shimadzu GC‐2030/QP2020 NX) equipped with an SP‐2340 fused‐silica capillary column (30 m × 0.25 mm × 0.20 μm). Helium was used as the carrier gas. Fatty acids were identified by comparison with authentic external standards, and quantification was performed using C17:0 as an internal standard according to [25]. The results were expressed as a percentage of the total identified fatty acids.

#### 2.7.4. Antioxidant Capacity

Ground pellets (1 g) were extracted with 20 mL of 80% (v/v) methanol by ultrasound‐assisted extraction (30 min at 25°C). The extracts were centrifuged (4000 rpm, 15 min) and filtered (0.45 µm). Antioxidant activity was determined using the DPPH radical‐scavenging assay [26]. Absorbance was measured at 517 nm after 30 min incubation in the dark. Results were calculated from a Trolox calibration curve (*R* ^2^ > 0.99) and expressed as mg Trolox equivalents (TE) per gram dry weight (dw).

#### 2.7.5. Total Phenolic Content (TPC)

TPC was determined using the Folin–Ciocalteu colorimetric assay [27]. Aliquots of methanolic extracts were reacted with the Folin–Ciocalteu reagent followed by sodium carbonate solution and incubated in the dark (2 h, room temperature). The absorbance was measured at 765 nm. TPC was quantified using a gallic acid calibration curve (*R* ^2^ > 0.99) and expressed as mg gallic acid equivalents (GAE) per gram dw.

#### 2.7.6. Peroxide Value (PV)

The PV was determined as an indicator of primary lipid oxidation following a modified AOCS Official Method Cd 8b‐90 [28]. Lipid extracts were dissolved in acetic acid–chloroform (3:2, v/v) and reacted with potassium iodide, and the liberated iodine was titrated with standardized sodium thiosulfate solution using starch as an endpoint indicator. PV was calculated and expressed as milliequivalents of active oxygen per kilogram of extracted oil (meq O_2_ kg^−1^ oil).

#### 2.7.7. Water Activity (aw)

aw was measured at 25°C using a calibrated aw meter (AquaLab 4TE). The instrument was calibrated prior to analysis using certified salt standards (aw 0.25–0.76). Samples were equilibrated to room temperature and analyzed in sealed sample cups until stable readings were obtained.

### 2.8. Pellet Technological Stability and Structural Integrity

#### 2.8.1. Pellet Durability Index (PDI)

Pellet durability was determined as PDI (%), using a tumbling abrasion test. Briefly, a representative pellet fraction was sieved to remove pre‐existing fines (<1.0 mm), and a fixed mass of pellets (100 g) was placed in a pellet durability tester. Samples were tumbled for 10 min at 50 rpm. After tumbling, pellets were resieved using the same sieve to remove the fines generated, and the retained pellet mass was recorded. PDI was calculated as follows:

(4) PDI %=mretainedminitial⋅100.

#### 2.8.2. Pellet Hardness (Compression Test)

Pellet hardness (N) was measured using a texture/compression test on individual pellets. Pellets were equilibrated to room temperature, and 20 pellets per replicate were tested using a texture analyzer (Omnitest‐50, Mecmesin, UK) fitted with a flat compression probe. Each pellet was placed horizontally on the platform and compressed at a constant crosshead speed (1.0 mm s^−1^) until fracture. The maximum force at break (N) was recorded as the pellet hardness.

#### 2.8.3. Fines Generation

Fines (%) were quantified gravimetrically by sieving. A known mass of pellets (100 g) was sieved for 2 min using a 1.0 mm mesh to remove fine particles generated during storage/handling. The mass of fines collected below the sieve was measured and expressed as a percentage of the initial sample mass:

(5) Fines %=mfinesminitial ⋅100.

#### 2.8.4. Water Stability (60 min; % Retained)

Water stability was assessed as the percentage of pellet mass retained after 60 min of immersion in water. Pellets were sieved to remove pre‐existing fines (<1.0 mm), and a fixed mass (100 g) was placed in a beaker containing 200 mL of deionized water at 25 ± 1°C, under mild agitation (100 rpm). After 60 min, the remaining pellet solids were recovered using a sieve (e.g., 0.5–1.0 mm), briefly rinsed to remove adherent fragments, and dried at 105°C to a constant mass. Water stability was expressed as follows:

(6) Water stability % retained=mdry,retainedmdry,initial⋅100.

All analyses were performed in triplicate (*n* = 3) at each sampling point, and results are reported as the mean ± standard deviation.

### 2.9. Statistical Analysis

All experimental results are expressed as the mean ± standard deviation (*n* = 3). Statistical analyses were performed using IBM SPSS Statistics (Version 27.0, IBM Corp., Armonk, NY, USA). Data were assessed for normality and homogeneity of variance prior to the analysis. Two‐way analysis of variance (ANOVA) with interaction was applied to evaluate the effects of storage time and feed formulation on physicochemical composition, oxidative stability parameters, and pellet technological properties. The interaction term (time × formulation) was used to determine whether storage effects differed among formulations. When interaction effects were not statistically significant (*p*  > 0.05), main effects were interpreted independently. When significant differences were detected (*p*  < 0.05), Tukey’s post hoc test was used for pairwise comparisons between treatments. Correlation analysis among physicochemical and technological parameters was performed using the Spearman’s rank correlation coefficient (*ρ*) because the observed relationships were monotonic. Data visualization and graphical representation were carried out using OriginPro 8.5 (OriginLab Corporation, Northampton, MA, USA). Statistical significance was set at α = 0.05. To improve statistical transparency while maintaining readability of the main text, two‐way ANOVA outputs for selected key parameters, including moisture content, aw, PV, and PDI, are provided in the Supporting Information.

## 3. Results and Discussion

### 3.1. Characterization of EEO

**Figure 1:**
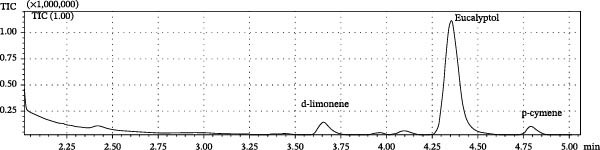
GC–MS chromatogram for eucalyptus essential oil showing the major constituents.

The chemical composition of EEO was determined by GC–MS, with eucalyptol (1,8‐cineole) identified as the dominant compound, accounting for 68.9% ± 0.27% (relative area) of the oil. Minor constituents included d‐limonene (1.53% ± 0.08%) and p‐cymene (1.01% ± 0.07%) (Figure 1). These findings are in agreement with previous studies, which consistently report eucalyptol as the principal component of *Eucalyptus globulus* oil, typically ranging between 60% and 85% depending on geographic origin and extraction conditions [29, 30].

Given the high content of eucalyptol, EEO demonstrates considerable potential as a functional additive in aquafeeds, particularly for its antimicrobial, antioxidant, and anti‐inflammatory properties [31]. These bioactivities are associated with enhanced gut health, improved immune response, and increased disease resistance in fish. Incorporation of eucalyptus oil has been reported to stimulate nonspecific immune parameters such as lysozyme activity, phagocytosis, and respiratory burst [32]. Therefore, the profile characterized for eucalyptus EO supports its selection as a model phytobiotic compound in the present study.

### 3.2. Preliminary Formulation Screening of Ulvan–Lecithin Microstructures

Preliminary formulation screening showed that lecithin incorporation influenced both the encapsulation performance and the physical properties of the ulvan‐based EEO microstructures (Table 2). The formulation prepared without lecithin (U–EEO) exhibited the lowest EE% (60.3% ± 2.8%) and the highest surface‐associated EEO fraction (39.7% ± 2.8% of total EEO), together with the largest median particle size (*d*_50_ = 186.4 ± 8.9 μm), the broadest size distribution (span = 1.75 ± 0.08), and a less negative ζ‐potential (−24.9 ± 1.5 mV). This suggests that, under the applied emulsification and freeze‐drying conditions, the ulvan matrix alone provided limited retention of the hydrophobic oil phase.

**Table 2:** Preliminary formulation screening of ulvan‐based eucalyptus essential oil microstructures prepared with different lecithin levels.

Formulation	EE (%)	Surface‐associated EEO (% of total EEO)	*d*_50_ (μm)	Span	z‐potential (mV)
U–EEO	60.3 ± 2.8	39.7 ± 2.8	186.4 ± 8.9	1.75 ± 0.08	−24.9 ± 1.5
U–L0.5–EEO	70.9 ± 1.9	29.1 ± 1.9	158.6 ± 6.4	1.47 ± 0.05	−29.0 ± 1.6
**U–L1–EEO**	**76.4 ± 1.2**	**23.6 ± 1.2**	**145.2 ± 4.8**	**1.33 ± 0.04**	**−31.6 ± 1.8**
U–L2–EEO	72.6 ± 1.7	27.4 ± 1.7	166.8 ± 7.2	1.52 ± 0.07	−34.1 ± 1.9

*Note:* Bold values indicate the selected formulation (U–L1–EEO), which exhibited the highest encapsulation efficiency and the lowest surface‐associated EEO, particle size (*d*_50_), and span among the screened formulations.

This interpretation is consistent with previous studies showing that ulvan can contribute to the stabilization of oil‐in‐water systems, although the performance of such systems depends strongly on formulation composition, interfacial organization, particle‐size distribution, and surface charge (https://doi.org/10.1002/ffj.3519). Incorporation of lecithin improved the overall formulation profile, with U–L0.5–EEO showing increased EE (70.9 ± 1.9%), reduced surface‐associated EEO (29.1 ± 1.9%), smaller particle size (*d*_50_ = 158.6 ± 6.4 μm), narrower distribution (span = 1.47 ± 0.05), and a more negative ζ‐potential (−29.0 ± 1.6 mV). This behavior is consistent with the amphiphilic character of lecithin, which has been widely used as a natural emulsifier for hydrophobic oil phases and has been reported to influence EE%, droplet dispersion, and oxidative stability in oil‐loaded microencapsulation systems [13, 33]. Among the screened formulations, U–L1–EEO provided the most favorable combined technological profile, exhibiting the highest EE (76.4 ± 1.2%), the lowest surface‐associated EEO fraction (23.6 ± 1.2%), the smallest *d*_50_ (145.2 ± 4.8 μm), the narrowest span (1.33 ± 0.04), and a ζ‐potential of −31.6 ± 1.8 mV. The latter value is within the range commonly associated with improved electrostatic stabilization in dispersed systems; however, in the present freeze‐dried microparticles, ζ‐potential should be interpreted mainly as an indicator of surface charge after redispersion rather than as direct evidence of long‐term colloidal stability. Increasing the lecithin concentration to 2% did not further improve encapsulation performance, despite producing a more negative ζ‐potential (−34.1 ± 1.9 mV). Instead, U–L2–EEO showed a slightly lower EE (72.6 ± 1.7%), a higher surface‐associated EEO (27.4 ± 1.7%), a larger particle size (d50 = 166.8 ± 7.2 μm), and a broader size distribution (span = 1.52 ± 0.07) than U–L1–EEO. This suggests that formulation selection should not be based on surface charge alone, but rather on the combined balance between oil retention, surface‐associated oil, particle‐size suitability, and redispersion behavior. The reduced performance at the highest lecithin level may reflect less favorable matrix organization or excess‐emulsifier effects during dehydration, although this mechanism was not directly investigated. Thus, the inclusion of the U–EEO formulation enabled assessment of the ulvan‐only matrix, while the lecithin‐gradient formulations allowed evaluation of lecithin’s contribution through changes in EE%, surface‐associated EEO, particle‐size distribution, and z‐potential. This design was used to support the rationale for selecting the combined ulvan–lecithin system rather than relying on a single final formulation without formulation screening. Although this screening does not represent an exhaustive formulation optimization, based on the comparative screening of all formulations in terms of EE%, surface‐associated EEO, particle‐size distribution, and ζ‐potential, U–L1–EEO was selected for subsequent in‐depth physicochemical and functional evaluation, including FTIR, DSC, release kinetics, simulated gastrointestinal digestion, and aquafeed incorporation studies. Therefore, comparison between U–EEO and the lecithin‐containing formulations enabled assessment of lecithin’s contribution under otherwise identical preparation conditions, while the lecithin gradient design facilitated selection of the optimal lecithin level.

### 3.3. Characterization of the Selected U–L1–EEO Microstructures

Following the preliminary formulation screening, U–L1–EEO was selected for in‐depth characterization because it provided the most favorable combined profile in terms of EE%, surface‐associated EEO, particle‐size distribution, and ζ‐potential. The EE% of EEO within the selected ulvan–lecithin matrix reached 76.4% ± 1.2%, indicating effective retention of the lipophilic active compound through the emulsion‐assisted freeze‐drying process. This value is within the range reported for essential oils encapsulated in natural polysaccharide‐based systems, including chitosan‐ and alginate‐based matrices [34–36]. The relatively high EE observed for U–L1–EEO may be associated with the combined contribution of lecithin‐mediated emulsification, which can support the dispersion of hydrophobic oil droplets within the aqueous ulvan phase, and freeze‐drying, which avoids prolonged exposure to elevated temperatures and may therefore reduce volatilization losses of monoterpenoid constituents. The amphiphilic nature of lecithin likely contributed to interfacial stabilization of the oil phase, limiting coalescence and surface migration of the essential oil during dehydration. However, since pre‐drying emulsion droplet size and interfacial properties were not directly measured, this interpretation should be considered literature‐supported rather than direct mechanistic proof. Microencapsulation has also been applied to oil‐rich feed‐related ingredients, such as spray‐dried menhaden fish oil, supporting the broader relevance of encapsulation strategies for the processing and handling of hydrophobic bioactives in feed systems [37]. This processing advantage is particularly relevant for volatile phytobiotics such as EEO. From an application perspective, the obtained EE suggests that a substantial fraction of the added phytobiotic was retained within the dried microstructure, supporting further evaluation of release kinetics and feed stability. Essential oils have been reported to enhance antioxidant capacity, immune response, and disease resistance in fish, although such biological effects remain to be confirmed for the present formulation through in vivo feeding trials [38–40]. Overall, the obtained EE% confirms the suitability of the ulvan–lecithin matrix as a protective carrier for volatile bioactives and establishes a solid foundation for subsequent evaluation of release kinetics and feed stability.

### 3.4. Particle Size Distribution

**Figure 2:**
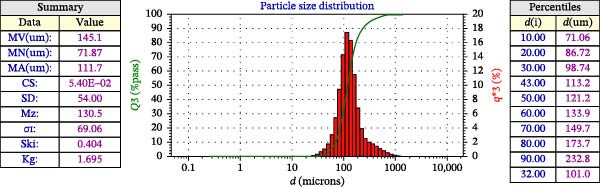
Particle size distribution of ulvan–lecithin microencapsulated eucalyptus essential oil, as determined by laser diffraction.

Particle size is a critical parameter for the successful application of microencapsulated additives in aquafeeds, as it may influence powder handling, feed homogeneity, pellet integrity, release behavior, and gastrointestinal fate. In food and feed systems, microencapsulated carriers are commonly reported in the micrometer range, from tens to a few 100 micrometers, which is generally considered suitable for incorporation into compound feeds [41]. In the present study, the selected U–L1–EEO microstructures exhibited a median particle diameter (*d*_50_) of ~145 μm, with *d*_10_ ≈ 71 μm and *d*_90_ ≈ 233 μm, yielding a span of 1.33 (Figure 2). This indicates a moderately narrow particle‐size distribution and is consistent with values reported for polysaccharide‐based microparticles intended for food and feed applications [41, 42].

From a technological perspective, particle sizes below ~250–300 μm are compatible with the granulometry of ground aquafeed ingredients, which are typically milled to support homogeneous mixing and cohesive pellet formation [43]. Accordingly, the average particle size of ~145 μm suggests that the selected microstructure is suitable for incorporation into the feed matrix without major size‐related segregation. The particle size is also substantially smaller than the diameter of the commercial aquafeed pellets used in this study (1.7 mm), supporting its compatibility with pellet incorporation [44]. Nevertheless, homogeneous distribution in the final feed cannot be inferred from particle‐size measurements alone and should be interpreted alongside the subsequent pellet stability results. The relatively narrow size distribution may also contribute to more predictable release behavior by reducing the variability in diffusion path length. From a physiological perspective, microparticles in the range of several tens to a few hundred micrometers are expected to remain within the gastrointestinal lumen and provide localized release of active compounds, whereas nanoscale carriers may exhibit faster release kinetics and increased tissue penetration [45, 46]. In this context, the size characteristics of U–L1–EEO represent a suitable compromise between loading capacity, feed compatibility, and controlled‐release potential.

### 3.5. Zeta‐Potential

The ζ‐potential of the selected U–L1–EEO microstructure was −31.6 ± 1.8 mV after redispersion in water, indicating a negatively charged particle surface. Absolute ζ‐potential values greater than ~30 mV are commonly associated with improved electrostatic stabilization in dispersed systems due to repulsive interactions between particles [47]. The negative charge observed in the present system is consistent with the presence of sulfated and carboxylated groups in ulvan, as well as polar phospholipid components from lecithin. Similar values have been reported for lecithin–polysaccharide microspheres (−30.86 mV) [48] and ulvan‐based emulsions exhibiting highly negative surface charge, approximately −46 mV [49], supporting the anionic character of marine polysaccharide‐based delivery systems.

However, in the present study, the ζ‐potential should be interpreted primarily as an indicator of the surface charge of redispersed microparticle fragments rather than as direct evidence of the long‐term colloidal stability of the dried powder or the final aquafeed matrix. Although electrostatic repulsion may contribute to dispersion behavior in dilute aqueous measurements, the practical stability of feed‐relevant microparticles in the 50–300 μm range is expected to depend strongly on matrix structure, hydration behavior, and physical integrity, in addition to surface charge. In aquafeed applications, such surface‐charge characteristics are therefore most relevant to dispersion behavior during mixing and release testing, while the technological performance of the system should be assessed through the subsequent feed stability parameters. Overall, the EE, particle‐size distribution, and ζ‐potential of the selected U–L1–EEO microstructure support its suitability for further investigation as a phytobiotic carrier in aquafeed applications.

### 3.6. Evaluation of FTIR Spectra

**Figure 3:**
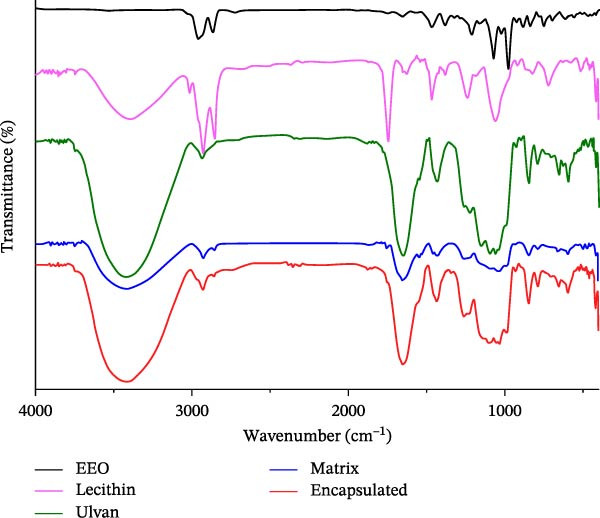
FTIR spectra of lecithin, ulvan, ulvan–lecithin matrix, and ulvan–lecithin microparticles containing eucalyptus essential oil.

FTIR spectroscopy was employed to investigate potential intermolecular interactions within the ulvan–lecithin encapsulation system and to verify the incorporation of EEO (Figure 3). Pure ulvan exhibited the characteristic absorption bands of sulfated polysaccharides, including a broad –OH stretching band around 3400 cm^−1^, C–H stretching near 2920 cm^−1^, asymmetric and symmetric carboxylate (COO^−^) vibrations around 1600–1630 cm^−1^, sulfate ester (S = O) stretching at ~1220–1260 cm^−1^, and glycosidic C–O–C vibrations near 1030–1070 cm^−1^, confirming preservation of its structural backbone. Lecithin displayed typical phospholipid features, including C–H stretching vibrations (2925–2850 cm^−1^), ester carbonyl absorption (~1735 cm^−1^), and phosphate‐related bands in the 1245–1255 cm^−1^ region, consistent with previous reports [50]. Pure EEO showed characteristic bands associated with monoterpenoid constituents, particularly eucalyptol, including aliphatic C–H stretching vibrations in the 2960–2850 cm^−1^ region, C–H bending vibrations around 1460–1370 cm^−1^, and C–O–C/C–O stretching bands in the 1200–1000 cm^−1^ region, in agreement with previous FTIR‐based essential oil characterization studies [51, 52]. Upon formation of the ulvan–lecithin matrix, the main characteristic peaks of both components were retained; however, slight shifts (5–15 cm^−1^) and intensity changes were observed in the –OH, COO^−^, and phosphate/sulfate regions. In particular, the broadening and minor displacement of the –OH band, together with modifications in the 1200–1250 cm^−1^ region, suggest the establishment of hydrogen bonding and electrostatic interactions between the charged sulfate groups of ulvan and the polar phospholipid headgroups of lecithin. No new absorption bands were detected, indicating that the interaction is predominantly physical rather than covalent in nature [53]. In the final system containing EEO, further broadening of the –OH band (~3400 cm^−1^) and enhancement of the C–H stretching bands (~2925 cm^−1^) were observed, attributable to the oil’s monoterpenoid constituents, predominantly eucalyptol. Concurrent attenuation of certain polar absorptions is consistent with the partial shielding of hydrophilic groups and the incorporation of the lipophilic oil into the matrix interior. Similar spectral behavior has been reported for essential oil encapsulation in polysaccharide‐based systems, where retention of matrix bands, peak broadening, attenuation, or overlap of essential‐oil signals were associated with physical incorporation and noncovalent interactions rather than covalent bonding [54, 55]. Overall, the FTIR results support the formation of a physically stabilized ulvan–lecithin network capable of incorporating lipophilic active compounds through non‐covalent interactions, providing structural evidence for the observed release and stability behavior.

### 3.7. Evaluation of DSC Results

**Figure 4:**
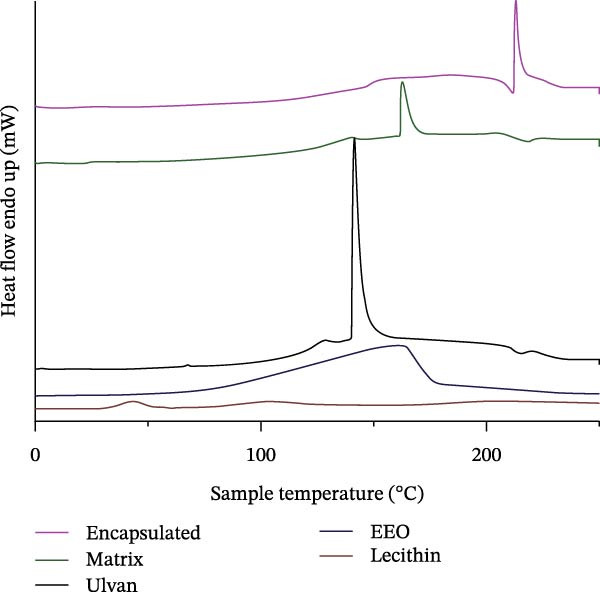
DSC thermograms of lecithin, pure eucalyptus essential oil (EEO), ulvan, unloaded ulvan–lecithin matrix, and selected U–L1–EEO microstructures containing eucalyptus essential oil. Endothermic direction is upward.

DSC was employed to evaluate the thermal behavior of pure EEO, ulvan, lecithin, the unloaded ulvan–lecithin matrix, and the selected U–L1–EEO microstructures. For the polymer‐containing samples, glass‐transition temperatures (Tg) were determined from the maximum of the first derivative of the heat flow signal during the second heating cycle to eliminate thermal history effects (Figure 4). Pure EEO was included as a reference to distinguish the thermal behavior of the volatile oil phase from that of the polymeric ulvan–lecithin matrix. As EEO is a liquid mixture rich in volatile monoterpenes, its DSC profile was interpreted mainly in relation to the volatilization‐associated endothermic behavior rather than glass transition phenomena. Pure ulvan exhibited a Tg value at ~140°C, consistent with reported values for sulfated polysaccharides, where Tg is influenced by molecular weight, ionic substitution, and residual moisture content [56]. A secondary thermal event observed near 250°C is assigned to the onset of thermal decomposition, as commonly reported for hydrophilic polysaccharide matrices [57]. Upon lecithin incorporation, the ulvan–lecithin matrix exhibited an increased Tg (163°C). The elevation of Tg indicates reduced segmental mobility of the polymer chains, likely due to intermolecular interactions between the ulvan and lecithin components. Such interactions may reduce the free volume and restrict molecular motion within the network, thereby enhancing thermal stability. Comparable increases in Tg have been reported in polysaccharide–phospholipid composite systems [58–60]. In contrast, the incorporation of EEO into the ulvan–lecithin matrix decreased Tg to ~123°C. This decrease may indicate a plasticizing‐like effect of low‐molecular‐weight EEO constituents within the ulvan–lecithin matrix, leading to increased molecular mobility. However, because pure EEO also exhibits volatilization‐associated thermal behavior, this transition should be interpreted cautiously as an apparent matrix‐transition shift rather than as direct proof of a specific plasticization mechanism.

Similar decreases in matrix thermal transitions after essential oil incorporation have been observed in biopolymer‐based encapsulation systems and have been associated with plasticizing effects or partial modification of polymer–polymer interactions [61, 62]. The free EEO thermogram showed a broad thermal event consistent with the volatilization behavior expected for essential‐oil constituents. A similar thermal behavior has been reported for essential‐oil systems, where endothermic events are associated with evaporation or volatilization of the free oil phase [63]. In contrast, the selected U–L1–EEO thermogram did not show a distinct free‐oil thermal event of comparable intensity, but was instead dominated by the thermal behavior of the ulvan–lecithin matrix. The attenuation or masking of the free‐oil thermal signature after encapsulation supports physical incorporation of EEO within the matrix, in agreement with previous essential‐oil microencapsulation studies in which the disappearance or reduction of guest‐oil thermal events was associated with successful encapsulation and dominance of the wall material thermal profile [63, 64]. This interpretation is also consistent with the reduced surface‐associated EEO measured for U–L1–EEO and with FTIR evidence indicating primarily noncovalent interactions among ulvan, lecithin, and EEO. From a practical standpoint, the main matrix‐associated thermal transitions occurred at or near the thermal range relevant to feed conditioning, suggesting that the developed microstructures exhibited thermal behavior compatible with the processing conditions evaluated in this study. The observed combination of increased Tg upon lecithin incorporation and a decrease in apparent transition temperature upon oil loading reflects a balance between matrix rigidity and flexibility, which may contribute to both structural integrity and controlled‐release performance in aquafeed applications [65].

### 3.8. Release Kinetics Study

**Figure 5:**
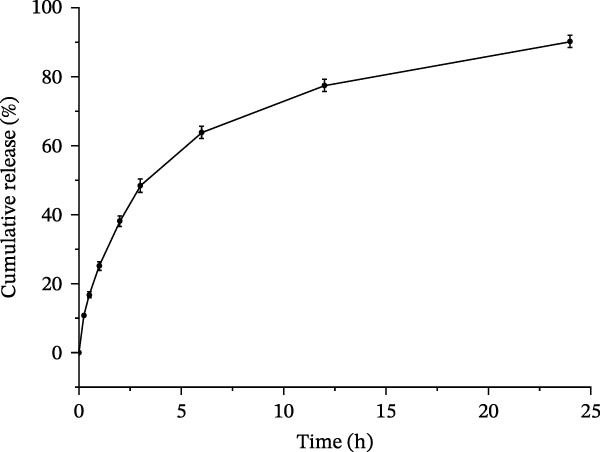
Cumulative release profile of eucalyptus essential oil from ulvan–lecithin microparticles in PBS (pH 7.4).

The release profile of EEO from ulvan–lecithin microparticles in PBS (pH 7.4) exhibited a biphasic behavior, characterized by an initial faster release phase, followed by a progressively slower, sustained release period (Figure 5). Approximately 25% of the encapsulated oil was released within the first hour, increasing to ~64% at 6 h and reaching ~90% cumulative release after 24 h. Although an initial acceleration in release was observed, the absence of an extreme burst effect (i.e., rapid release exceeding 40%–50% within the first hour) suggests effective incorporation of the essential oil within the polysaccharide matrix and limited surface‐associated oil. The early release stage is attributed to the fraction of oil located near or within the microparticles’ outer hydrated regions. Upon contact with the aqueous medium, rapid hydration of the ulvan–lecithin network facilitates the diffusion of this accessible fraction. Subsequently, the release rate decreases as transport becomes controlled by diffusion through the progressively hydrated polymer matrix. Comparable biphasic release profiles have been reported for essential oils encapsulated in alginate and chitosan‐based systems [66, 67].

Kinetic modeling was applied to further elucidate the release mechanism (Table 3).

**Table 3:** Kinetic parameters and goodness‐of‐fit indices for the release of eucalyptus essential oil from ulvan–lecithin microparticles in PBS.

Model	Parameters	Value	*R* ^2^	RMSE
Zero order	*k*_0_ (h^−1^)	4.850	0.786	23.41
First order	*k*_1_ (h^−1^)	0.094	0.786	14.98
Higuchi	*k*_H_ (%h^−1/2^)	21.477	0.975	7.92
Korsmeyer‐Peppas	*k*_KP_ (h^−*n*^), *n*	0.021, 0.602	0.999	0.13

Zero‐order and first‐order models exhibited limited agreement with the experimental data (*R* ^2^ = 0.786), indicating that the release did not proceed at a constant rate nor follow a purely concentration‐dependent exponential decay. In contrast, the Higuchi model provided a substantially improved fit (*R* ^2^ = 0.976), supporting diffusion through a hydrated matrix as the dominant release mechanism. The Korsmeyer–Peppas model, applied to the initial release stage (Rt < 60%), yielded an excellent fit (*R* ^2^ = 0.999; RMSE = 0.13), with a release exponent *n* = 0.602. For spherical matrices, values of 0.43 < *n* < 0.85 indicate anomalous (non‐Fickian) transport, where diffusion and polymer relaxation/swelling contribute simultaneously to mass transfer [20, 68].

Taken together, the combined Higuchi and Korsmeyer–Peppas analyses suggest that the release of EEO from ulvan–lecithin microparticles is predominantly diffusion‐controlled, with a secondary contribution from matrix relaxation phenomena during hydration. This interpretation is consistent with the FTIR and DSC results, which indicated the formation of a physically stabilized, hydration‐responsive polysaccharide–phospholipid network. From an application perspective, the sustained‐release profile observed in PBS supports the suitability of the developed microparticles for incorporation into aquafeed, where controlled availability of phytobiotics during feed immersion and ingestion is desirable to limit premature losses and enhance bioactive persistence.

#### 3.8.1. In Vitro Digestion Under Simulated *Dicentrarchus labrax* Conditions

**Figure 6:**
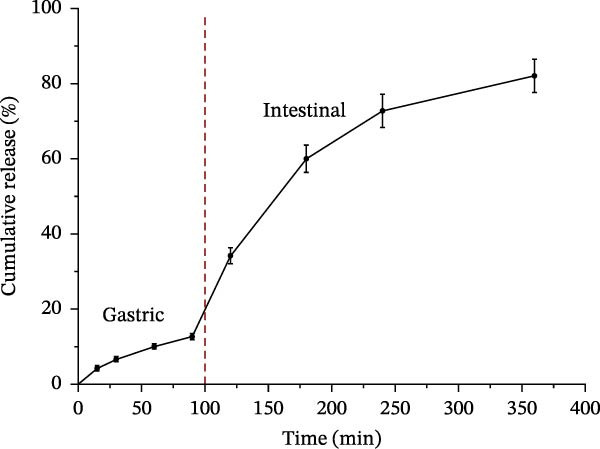
In vitro gastrointestinal cumulative release profile of eucalyptus essential oil from U–L1–EEO. The dashed vertical line indicates the transition from gastric conditions (pH 2.5) to intestinal conditions (pH 7.5 with pancreatin and bile salts) after 90 min of gastric incubation.

The in vitro gastrointestinal release profile of EEO from ulvan–lecithin microparticles exhibited a clear phase‐dependent behavior, with limited release under gastric conditions and pronounced release following transition to intestinal conditions (Figure 6). During the gastric phase (0–90 min, pH 2.5), cumulative release increased gradually to 12.7%, indicating strong retention of the phytobiotic within the matrix under acidic conditions.

Upon transition to intestinal conditions, cumulative release increased from 12.7% to 34.2% at the first intestinal sampling point (120 min total digestion time) and reached 82.0% at 360 min total digestion time. Thus, ~69.3% of the encapsulated oil was liberated during the intestinal phase alone. These results suggest that only a minor fraction of the payload was released in the gastric environment, whereas the majority became available under intestinal conditions, likely due to enhanced matrix hydration, increased solubilization of lipophilic components by bile salt micelles, and partial structural weakening of the hydrated matrix. Taken together, the simulated *Dicentrarchus labrax* digestion results suggest that the ulvan–lecithin system limited premature release under acidic gastric conditions while enabling substantial release in the intestinal environment under the applied in vitro conditions.

Kinetic modeling was applied separately to each phase (Table 4). For the gastric phase, the Higuchi model provided an excellent fit (*R* ^2^ = 0.997; RMSE = 0.41), indicating diffusion‐controlled transport through the partially hydrated matrix. The Korsmeyer–Peppas model, applied to the initial gastric release region, also yielded an excellent fit (*R* ^2^ = 0.999; RMSE = 0.079) with a release exponent *n* = 0.604, consistent with anomalous transport where diffusion is coupled with matrix relaxation or swelling phenomena.

**Table 4:** Phase‐dependent release metrics and kinetic modeling parameters for the in vitro gastrointestinal release of eucalyptus essential oil.

Phase metrics				
Phase	Time range (min)	Cumulative release at phase end (%)	Mean rate (%•h^−1^)	
Gastric	0–90	12.7 ± 0.8	8.47	
Intestinal	90–360	82.0 ± 4.9 (+69.3)	15.4	

**Kinetic modeling per phase**				
**Phase**	**Model**	**Parameters**	***R* ^2^**	**RMSE**

Gastric	Higuchi	*k*_H_ = 10.01% h^−1/2^	0.997	0.41
Gastric	Korsmeyer‐Peppas	*k*_KP_ = 0.100 h^−*n*^, *n* = 0.604	0.999	0.079
Intestinal	First‐order (to Rmax)	*k* = 0.707, Rmax = 85.0%	0.999	0.026

In contrast, the intestinal phase was better described by an asymptotic first‐order model approaching a maximum releasable fraction (Rmax ≈ 85%), with a rate constant *k* = 0.707 h^−1^. This behavior reflects a shift from diffusion‐limited release to a solubilization‐enhanced regime, in which higher pH, bile salt micellization, and enzymatic activity collectively increase oil dispersion and facilitate transport from the matrix.

### 3.9. Storage Stability of Phytobiotic‐Enriched Aquafeeds

#### 3.9.1. Moisture Content and Water Activity

**Figure 7:**
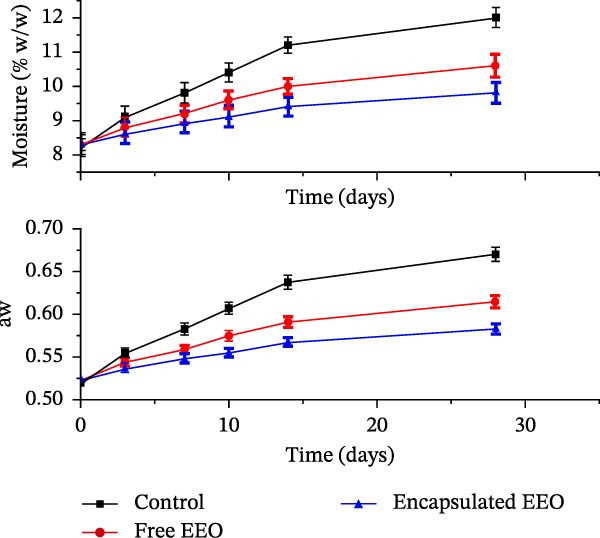
Evolution of moisture content (% w/w) and water activity (*a*_w_) of control, free EEO, and encapsulated EEO aquafeeds during accelerated storage (45°C, 70% RH).

Moisture content increased progressively during accelerated storage in all formulations (Figure 7), confirming the hygroscopic behavior of compound aquafeed pellets exposed to elevated temperature and humidity [69]. However, the magnitude of the change was strongly dependent on formulation. The control exhibited the largest increase, rising from 8.2% to 12.0% w/w (+46.3%), whereas EEO‐supplemented diets showed a significantly lower moisture uptake. The encapsulated formulation increased from 8.3% to 9.8% (+18.1%), corresponding to a ~61% reduction in moisture gain relative to the control. Two‐way ANOVA revealed significant effects of formulation, storage time, and their interaction on moisture content (*p*  < 0.05; Table S3), indicating that moisture uptake was affected by both feed formulation and storage duration, and that the magnitude of the increase in moisture over time differed among formulations. Post hoc comparisons revealed no significant differences between formulations at the initial storage stages (days 0–3; *p*  > 0.05), indicating comparable initial moisture behavior. However, from day 7 onwards, a progressive statistical separation emerged. At intermediate stages, the control exhibited significantly higher moisture levels than the encapsulated formulation, whereas the free EEO treatment showed an intermediate behavior without consistent statistical differentiation. By day 28, all formulations were significantly different (*p*  < 0.05), establishing a clear hierarchy in moisture uptake (Control > Free EEO > Encapsulated EEO) (Table S4). The reduced water uptake observed in EEO‐containing diets may be partly associated with the hydrophobic nature of essential oil constituents and their distribution within the pellet matrix, which has been suggested in the literature to influence water diffusion in food and feed systems [70]. In the encapsulated formulation, hydrophobic oil domains within the ulvan–lecithin microstructure may have contributed to the lower moisture uptake; however, this interpretation should be considered a literature‐supported hypothesis rather than a directly verified mechanism, as water diffusion, surface hydrophobicity, and barrier properties were not directly measured in the present study. A similar statistically significant trend was observed for aw, with two‐way ANOVA confirming significant effects of formulation, storage time, and their interaction (*p*  < 0.05; Table S5). Consistent with moisture content, no significant differences between treatments were observed at the initial stages (days 0–3; *p*  > 0.05), while a gradual separation emerged during storage. At intermediate stages, the control exhibited significantly higher aw values than the encapsulated formulation, whereas the free EEO treatment showed an intermediate behavior. From day 10 onwards, all formulations were significantly different (*p*  < 0.05), with the encapsulated system consistently maintaining the lowest aw values (Table S6). The maintenance of low moisture content and aw is critical for feed stability, as elevated humidity promotes microbial proliferation and accelerates oxidative and enzymatic deterioration. Moisture levels below ~10% are generally considered desirable for the prolonged shelf life of compound feeds [71]. Notably, the control formulation exceeded this threshold during storage, whereas the encapsulated EEO diet remained the closest to it throughout the 28‐day period, indicating improved resistance to environmental moisture ingress.

#### 3.9.2. Primary Lipid Oxidation and Lipid Retention

**Figure 8:**
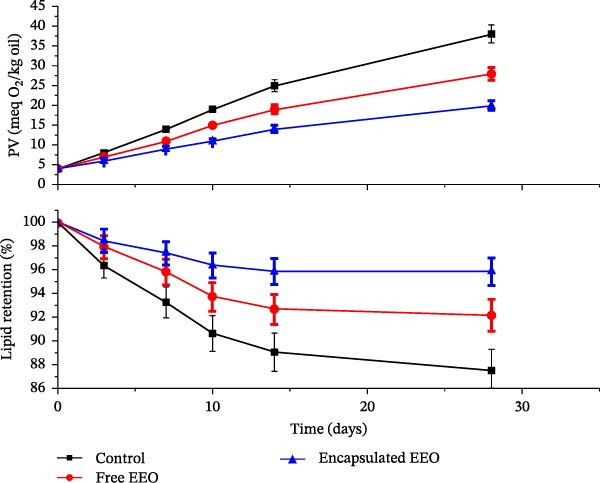
Evolution of peroxide value (PV) and lipid retention (%) of control, free EEO, and encapsulated EEO aquafeeds during accelerated storage (45°C, 70% RH). Values are expressed as mean ± SD (*n* = 3).

The PV, an indicator of primary lipid oxidation, increased progressively during storage across all treatments, reflecting the intrinsic susceptibility of fish oil polyunsaturated fatty acids to oxidative degradation (Figure 8). This increase was statistically significant over time for all formulations (*p*  < 0.05). However, oxidative progression was strongly dependent on the formulation. The control exhibited the most pronounced increase (4 → 38 meq O_2_ kg^−1^ oil), whereas both EEO‐supplemented diets showed significantly slower PV development, with the encapsulated formulation reaching only ~20 meq O_2_ kg^−1^ at day 28. Post‐hoc comparisons revealed that no significant differences between treatments were observed at the initial stage (day 0; *p*  > 0.05), while from day 3 onwards, all formulations were significantly different (*p*  < 0.05), following a consistent hierarchy (Control > Free EEO > Encapsulated EEO) throughout storage (Table S7). Two‐way ANOVA confirmed significant effects of formulation, storage time, and their interaction on PV values (*p*  < 0.05; Table S8), indicating that lipid oxidation progressed during accelerated storage and that the magnitude of PV increase differed among formulations. The lower PV values observed in the EEO‐supplemented formulations, particularly in the encapsulated system, are consistent with improved protection against lipid oxidation during storage. The magnitude of separation between treatments increased over time, indicating cumulative protection against lipid oxidation in the encapsulated system. Phenolic and terpenoid compounds are known to interrupt radical chain reactions and delay the propagation of lipid peroxidation [15, 72]. Consistent with PV trends, total lipid content decreased across all treatments, with the largest reduction observed in the control (−12.5%), compared with markedly lower losses in the free EEO (−6%) and encapsulated EEO (−4.17%) diets. Statistical analysis indicated no significant differences between formulations during the initial storage stages (days 0–7; *p*  > 0.05). On day 10, a partial separation emerged, with encapsulated samples exhibiting significantly higher lipid retention than the control, while the free EEO treatment showed an intermediate behavior. This trend became more pronounced at day 14, where both EEO‐supplemented diets differed significantly from the control (*p*  < 0.05). By day 28, all formulations were statistically distinct (Control < Free EEO < Encapsulated EEO), confirming a progressive enhancement in lipid preservation associated with encapsulation (Table S9). Preservation of the total lipid content was accompanied by improved retention of nutritionally critical ω−3 fractions. After 28 days, total ω−3 and EPA + DHA retention reached ~53% in the control, 64% in the free EEO diet, and ~75% in the encapsulated formulation, corresponding to a ~41% improvement in ω−3 preservation relative to the control. Consequently, the ω−3/ω−6 ratio declined from ~2.50 at day 0 to ~1.97 in the control, whereas higher ratios were maintained in EEO‐supplemented feeds (≈2.11 for free EEO; ≈2.24 for encapsulated EEO). These results are consistent with reduced oxidative degradation of long‐chain polyunsaturated fatty acids in the presence of protected phytochemical antioxidants. Overall, the encapsulated formulation exhibited superior preservation of lipid quality, suggesting that microencapsulation enhanced the stability and sustained activity of antioxidant compounds within the feed matrix during storage. The strong statistical agreement between PV development and lipid retention further supports lipid oxidation as the primary driver of nutritional degradation during storage.

#### 3.9.3. Antioxidant Capacity and TPC

**Figure 9:**
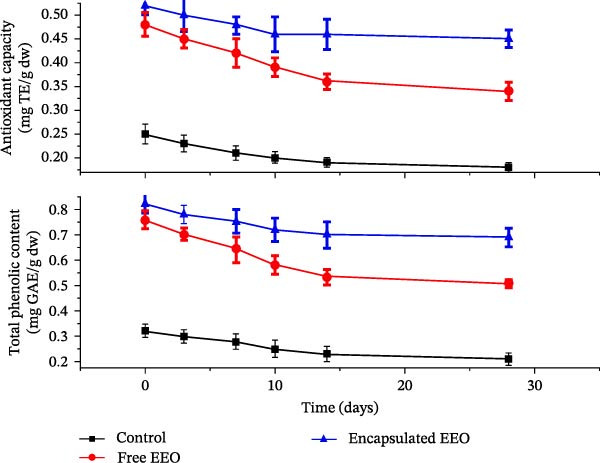
Changes in antioxidant capacity (mg TE g^−1^ dw) and total phenolic content (mg GAE g^−1^ dw) of control, free EEO, and encapsulated EEO aquafeeds during accelerated storage (45°C, 70% RH). Values represent mean ± SD (*n* = 3).

Initial antioxidant activity was substantially higher in EEO‐supplemented feeds (0.48–0.52 mg TE g^−1^) compared with the control (0.25 mg TE g^−1^), consistent with the presence of phenolic and monoterpenoid constituents naturally occurring in EEO [73]. During storage, antioxidant capacity declined progressively in the control and free EEO treatments, consistent with consumption or degradation of radical‐scavenging compounds under elevated temperatures and humidity [74] (Figure 9). This decline was treatment‐dependent. Post hoc comparisons showed that at the initial stage (days 0–3), both EEO‐supplemented diets exhibited significantly higher antioxidant capacity than the control, while no significant difference was observed between free and encapsulated EEO. From day 7 onwards, all formulations differed significantly (Encapsulated EEO > Free EEO > Control; *p*  < 0.05; Table S10). The encapsulated formulation showed improved antioxidant retention, with only ~13.5% loss after 28 days compared with ~28%–29% loss in the control and free EEO diets. Notably, within‐treatment comparisons indicated that antioxidant capacity in the encapsulated formulation remained statistically unchanged throughout storage, whereas significant declines were observed in both the control and free EEO diets (*p*  < 0.05). TPC followed a similar, although not identical, pattern. Initial phenolic levels were significantly higher in EEO‐containing feeds (0.76–0.82 mg GAE g^−1^) than in the control (0.32 mg GAE g^−1^). Although phenolic concentrations decreased during storage, this decline was likely associated with general degradation, volatilization‐related losses, and possible polymerization reactions under accelerated aging conditions, rather than oxidation alone [75]. Nevertheless, the magnitude of loss was substantially lower in the encapsulated formulation (≈−16%) relative to the control (≈−34%) and free EEO diet (≈−33%). Statistical analysis showed that on day 0, both EEO‐containing diets had significantly higher TPC than the control diet, with no significant difference between the free and encapsulated treatments. From day 3 onwards, all formulations were significantly different (encapsulated EEO > free EEO > control; *p*  < 0.05; Table S11). Within‐treatment comparisons further indicated that TPC decline in the encapsulated formulation emerged only at later storage stages, whereas reductions in the control and free EEO diets occurred earlier and were more pronounced. The improved retention of antioxidant capacity and TPC in the encapsulated formulation may reflect a combination of chemical antioxidant activity and physical protection effects. EEO constituents may contribute to radical‐scavenging activity, whereas the ulvan–lecithin matrix may help protect labile compounds by reducing volatilization, degradation, and direct exposure to oxygen or moisture during storage. Therefore, the observed antioxidant stability should not be attributed solely to the direct antioxidant action of EEO constituents. Rather, the results are consistent with a combined stabilization effect involving both phytochemical activity and matrix‐mediated protection under accelerated storage conditions. The relative contributions of these mechanisms were not quantified separately in the present study.

#### 3.9.4. Protein Stability

The protein content remained relatively stable throughout accelerated storage, with reductions of less than 4% across all treatments. These minor decreases may reflect limited storage‐related changes in the protein fraction, potentially associated with moisture exposure, matrix interactions, or general degradation processes under accelerated aging conditions [76, 77]. However, protein oxidation was not directly assessed in the present study, as specific markers such as protein carbonyls or amino acid oxidation products were not measured. Therefore, the observed changes should be interpreted as variations in crude protein content rather than direct evidence of protein oxidative damage. Compared with lipid and antioxidant parameters, the crude protein content was less affected by storage conditions, suggesting relative stability of the protein fraction under the applied aging regime. Formulation‐dependent differences were nevertheless observed, with EEO‐supplemented diets exhibiting slightly lower protein losses and the encapsulated formulation showing the smallest reduction (≈−1.65%). Preservation of protein content is relevant in aquafeeds as proteins provide essential amino acids required for growth performance and tissue maintenance in farmed fish [78]. Overall, these results indicate that incorporation of the phytobiotic systems did not compromise the crude protein content of the feed during storage. However, further targeted analyses would be required to determine whether encapsulation influences protein oxidation pathways, amino acid preservation, or molecular‐level protein quality.

#### 3.9.5. Pellet Technological Stability and Structural Integrity

**Figure 10:**
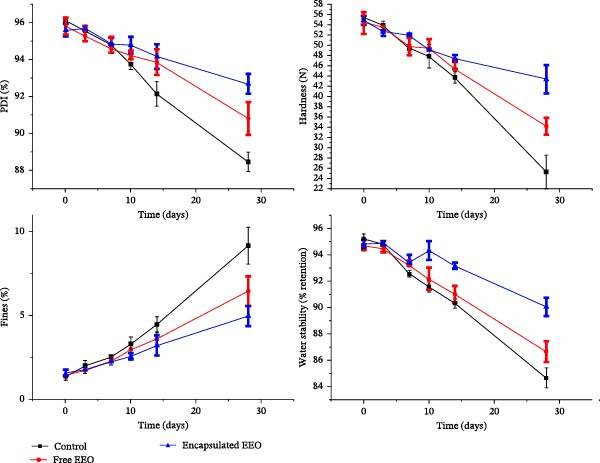
Evolution of pellet durability index (PDI), hardness (N), fines (%), and water stability (%) of control, free EEO, and encapsulated EEO diets during accelerated storage (45°C, 70% RH). Values are mean ± SD (*n* = 3).

Changes in pellet physical quality followed trends consistent with the observed chemical stability parameters (Figure 10). Mechanical durability (PDI) decreased in all formulations during storage, although the extent of deterioration was formulation‐dependent. The control exhibited a reduction of ~7.96%, whereas smaller losses were observed in the free EEO (−5.22%) and especially in the encapsulated EEO formulation (−3.04%). Two‐way ANOVA confirmed significant effects of formulation, storage time, and their interaction on PDI evolution (*p*  < 0.05; Table S12), indicating that pellet durability changed during accelerated storage and that the magnitude of this change differed among formulations. Post hoc comparisons indicated no significant differences between formulations during the early storage stages (days 0–7; *p*  > 0.05). From day 10 onwards, a progressive separation emerged, with encapsulated samples showing higher PDI values compared to the control, while the free EEO treatment exhibited intermediate behavior. By day 28, all formulations were significantly different (encapsulated EEO > free EEO > control; *p*  < 0.05; Table S13).

Hardness showed a more pronounced decline, decreasing by 54.4% in the control, by 35.8% in the free EEO, and by only 20.9% in the encapsulated formulation. The greater relative change in hardness than in PDI suggests that surface and internal structural weakening progressed during storage, even before complete pellet fragmentation became evident. Statistical analysis revealed that differences between formulations were not significant at early storage stages (days 0–10; *p*  > 0.05), whereas significant separation emerged at later time points. On day 14, encapsulated samples exhibited higher hardness than the control, while the free EEO treatment showed intermediate behavior. By day 28, the encapsulated formulation maintained significantly higher hardness than the control, while the free EEO treatment showed intermediate behavior (Table S14).

Fines generation increased markedly in all treatments, but the magnitude differed substantially: +563% in the control, +331% in the free EEO, and +215% in the encapsulated diet. However, statistical analysis indicated no significant differences between formulations up to day 14 (*p*  > 0.05). Only on day 28 did significant differences emerge, with the control exhibiting higher fine production than both EEO‐supplemented diets (*p*  < 0.05), while no significant difference was observed between the free and encapsulated formulations (Table S15). Similarly, water stability decreased by 11.0% in the control compared with 8.52% and 5.02% in the free and encapsulated formulations, respectively. Post hoc comparisons showed no significant differences in the early stages (days 0–7; *p*  > 0.05), whereas from day 10 onwards, encapsulated samples maintained significantly higher water stability than both the control and free EEO diets. By day 28, all formulations were significantly different (encapsulated EEO > free EEO > control; *p*  < 0.05; Table S16). Collectively, these results indicate that encapsulation progressively attenuated structural degradation during storage, with protective effects becoming more pronounced at later stages.

Correlation analysis further clarified the relationship between oxidative and technological deterioration. When data from all formulations and time points were pooled, PV exhibited a strong positive correlation with fines production (Spearman *ρ* = 0.973, *p*  < 0.001) and strong negative correlations with hardness (*ρ* = −0.944, *p*  < 0.001), PDI (*ρ* = −0.969, *p*  < 0.001), and water stability (*ρ* = −0.963, *p*  < 0.001). These correlations indicate that lipid oxidation and mechanical weakening progressed in parallel during accelerated storage. However, because correlation analysis does not establish causality, the results should not be interpreted as direct evidence that oxidation alone drove mechanical degradation. Rather, the observed relationships suggest that oxidative progression, moisture uptake, and structural weakening were closely associated components of the overall deterioration process. Formulations with lower PV development, particularly the encapsulated EEO diet, also maintained better pellet durability, hardness, and water stability, and exhibited lower fines formation during storage.

### 3.10. Overall Evaluation of Storage Performance

Correlation analysis provided further insights into the interdependence among physicochemical and technological stability parameters. When data from all formulations and storage times were considered collectively, moisture content exhibited a near‐perfect positive correlation with PV (*ρ* ≈ 0.99, *p*  < 0.001), indicating that both parameters followed closely aligned temporal trends during storage. However, these high correlations may partly reflect the shared effect of storage time rather than a direct causal relationship. Similarly, aw was highly positively correlated with PV (*ρ* ≈ 0.99), suggesting that moisture‐related changes and lipid oxidation developed in parallel under accelerated storage conditions [79].

The PV was strongly negatively correlated with total lipid retention (*ρ* ≈ −1.00), reflecting the expected inverse relationship between primary oxidation and remaining lipid content. This relationship should be interpreted cautiously, as lipid retention and oxidative deterioration are closely linked storage‐quality indicators and may be partially coupled. PV also showed strong negative correlations with antioxidant capacity (*ρ* ≈ −0.79) and total phenolics (*ρ* ≈ −0.87), consistent with progressive consumption or degradation of phytochemical antioxidants during storage.

Technological parameters were closely linked to oxidative indices. PV correlated positively with fines production and negatively with hardness, PDI, and water stability, indicating that oxidative deterioration was closely associated with the mechanical weakening of pellets. While correlation does not imply causation, the strength and consistency of these relationships suggest that moisture ingress, oxidative progression, and structural degradation form a tightly interconnected deterioration network during accelerated storage [80].

Within this network, the encapsulated EEO formulation consistently exhibited lower moisture uptake, reduced PV development, improved antioxidant preservation, and enhanced mechanical stability of pellets. These findings suggest that encapsulation was associated with improved stability across multiple interrelated quality parameters during accelerated storage. This protective behavior may involve both chemical and physical stabilization effects. The chemical contribution is supported by lower PV development and improved retention of antioxidant capacity and TPC, whereas the physical contribution is suggested by lower moisture uptake, reduced increase in aw, and improved pellet hardness, durability, water stability, and fines resistance. However, the relative contribution of each mechanism was not independently quantified. In particular, barrier properties, oxygen diffusion, water diffusion, and the separate effects of essential oil activity versus matrix‐mediated protection were not directly measured. Therefore, these results should be interpreted descriptively, reflecting concurrent improvements across several stability endpoints, rather than as direct mechanistic proof of individually resolved stabilization pathways. Overall, the relative changes recorded after 28 days of accelerated storage confirm the superior preservation of both chemical and technological quality in the encapsulated EEO formulation. The main physicochemical and oxidative stability changes are summarized in Table 5, while the corresponding changes in pellet physical quality parameters are summarized in Table 6.

**Table 5:** Relative changes (%) in physicochemical and oxidative stability parameters of experimental diets after 28 days of accelerated storage (45°C, 70% RH).

Parameter	Control	Free EEO	Encapsulated EEO
Moisture	+46.34	+27.71	+18.07
Aw	+28.85	+17.37	+11.26
Protein	−3.51	−2.48	−1.65
PV (meq O_2_/kg)	4 → 38	4 → 28	4 → 20
Lipids	−12.50	−7.85	−4.17
Omega‐3 retention	53	64	75
Antioxidant capacity	−28.0	−29.17	−13.46
Total phenolic content	−34.38	−32.89	−15.85

*Note:* Positive values indicate increases during storage; negative values indicate reductions relative to day 0.

**Table 6:** Relative changes (%) in pellet physical quality parameters after 28 days of accelerated storage (45°C, 70% RH).

Parameter	Control	Free EEO	Encapsulated EEO
PDI	−7.96	−5.22	−3.04
Hardness	−54.4	−35.8	−20.9
Fines	+563	+331	+215
Water stability	−11.0	−8.5	−5.0

*Note:* Positive values indicate increases during storage; negative values indicate reductions relative to day 0.

The improved stability may be associated with a combination of mechanisms, including reduced moisture ingress due to hydrophobic interactions; sustained antioxidant activity from the retention of protected phytochemicals; delayed lipid peroxidation; preservation of ω−3 fatty acids; and improved maintenance of pellet mechanical strength. This multi‐layer protective effect highlights the advantage of polysaccharide‐based microencapsulation strategies for delivering volatile phytobiotics in aquaculture feeds, where environmental exposure, oxidative stress, and mechanical handling simultaneously influence product quality.

### 3.11. Functional Implications for Aquafeed Performance

The integrated evaluation of release kinetics, simulated gastrointestinal behavior, and pellet technological properties suggests that the ulvan–lecithin microencapsulation system may contribute to both phytobiotic stabilization and phase‐selective release within aquafeed matrices. The limited release observed under simulated gastric conditions, followed by pronounced release in the simulated intestinal environment, suggests preferential intestinal release of EEO under the applied in vitro gastrointestinal conditions. This release profile may help limit premature release during gastric exposure and increase phytobiotic availability during the intestinal phase. In parallel, the encapsulated formulation was associated with improved oxidative stability, better preservation of antioxidant capacity and phenolic content, and reduced losses of polyunsaturated lipids during accelerated storage. These effects were accompanied by lower moisture uptake, improved mechanical durability, and reduced fines generation, indicating preservation of both nutritional and technological quality under storage stress. From a feed formulation perspective, these findings suggest that polysaccharide–phospholipid microstructures may support the incorporation of low‐inclusion volatile phytobiotics into aquafeeds by improving storage stability, limiting premature bioactive losses, and helping preserve pellet integrity during handling and storage. This could be relevant for commercial feed production, where additive retention, uniform dosing, reduced fines formation, and maintenance of pellet quality are important for product consistency. From a biological perspective, the preferential intestinal release observed under simulated gastrointestinal conditions may increase phytobiotic availability during the intestinal phase, with potential implications for antioxidant status, gut health, immune modulation, nutrient utilization, and growth performance. However, these application‐level benefits remain to be confirmed through in vivo feeding trials assessing intestinal release behavior, bioavailability, feed intake, growth performance, immune response, and fish health outcomes.

### 3.12. Industrial Translation and Future Validation

The present study provides a laboratory‐scale proof‐of‐concept evaluation of an ulvan–lecithin encapsulation system for delivering volatile phytobiotics in aquafeeds. Freeze‐drying was selected as a low‐temperature processing strategy to support oil retention and formation of a dry biopolymer matrix during initial formulation development, while limiting thermal stress on volatile EEO constituents. However, given the long processing times and relatively high energy demand associated with freeze‐drying [81], the present process should not be interpreted as an optimized industrial manufacturing route. Future scale‐up should compare the freeze‐dried system with more industrially scalable encapsulation technologies. In particular, spray drying is a continuous and widely used approach for oil and essential oil microencapsulation and can offer a more cost‐effective route for large‐scale feed applications [41, 82]. Future optimization should therefore assess whether comparable EE%, surface‐associated oil reduction, release behavior, feed‐processing tolerance, and storage‐stability benefits can be achieved under scalable processing conditions. Because production costs derived from laboratory‐scale freeze‐drying would not be representative of industrial manufacturing conditions, a reliable cost per kilogram of the encapsulated product was not calculated in the present study. A dedicated techno‐economic assessment based on scaled processing conditions, including raw material costs, energy consumption, process yield, drying capacity, labor, equipment depreciation, and production volume, will be necessary before commercial aquafeed implementation can be evaluated. Similarly, the static gastrointestinal simulation was designed for controlled comparative assessment of release behavior under fish‐relevant temperature conditions, rather than for exact replication of in vivo enzymatic degradation kinetics. In addition, although the in vitro release and simulated gastrointestinal assays provide controlled comparative evidence of pH‐ and digestion‐dependent EEO release, in vivo feeding trials are required to confirm bioavailability, feed intake, nutrient utilization, growth performance, immune response, gut health, and overall fish health outcomes under aquaculture‐relevant conditions [16, 83]. A further limitation of the present study is that direct GC–MS‐based mass balance quantification of EEO retention immediately before and after extrusion was not performed. Feed processing was conducted under industrial conditions at a collaborating feed‐production facility, which did not allow controlled immediate sampling, rapid quenching, and validated recovery of EEO at the extruder outlet. Therefore, EEO retention during extrusion and complete structural preservation of U–L1–EEO during processing cannot be conclusively confirmed. The present results should instead be interpreted as evidence of final‐pellet performance after incorporation and industrial processing, particularly during accelerated storage. Future work should include controlled pilot‐scale extrusion experiments with pre‐ and post‐extrusion GC–MS quantification, recovery validation, and structural characterization of the encapsulated system after processing.

## 4. Conclusions

This study indicates that ulvan–lecithin microstructures produced by emulsion‐assisted freeze‐drying may serve as a technically compatible carrier system for incorporating EEO into aquafeeds under the conditions evaluated. Preliminary formulation screening showed that incorporating lecithin improved the formulation profile compared with the ulvan‐only system, whereas increasing the lecithin concentration beyond 1% did not further enhance encapsulation performance. Among the screened systems, U–L1–EEO was selected as the most favorable formulation, combining the highest EE% (76.4% ± 1.2%), the lowest surface‐associated EEO fraction (23.6% ± 1.2%), suitable particle size for feed incorporation (*d*_50_ ≈ 145 μm), a relatively narrow size distribution, and a negative ζ‐potential after redispersion.

Physicochemical characterization supported the formation of a physically stabilized ulvan–lecithin matrix capable of incorporating the volatile oil phase mainly through non‐covalent interactions. FTIR analysis indicated interactions among ulvan, lecithin, and EEO without evidence of covalent bond formation, while DSC analysis, including pure EEO as a reference, supported physical incorporation of the oil within the matrix and matrix‐dominated thermal behavior. The main matrix‐associated transitions occurred within a thermal range relevant to the feed‐processing conditions evaluated in this study, indicating that thermal behavior may be relevant to processing performance.

Release studies indicated sustained and diffusion‐governed EEO release. In PBS, the selected formulation reached ~90% cumulative release after 24 h, with Higuchi and Korsmeyer–Peppas modeling supporting diffusion‐controlled and anomalous transport behavior during the initial release stage. Under simulated gastrointestinal conditions relevant to *Dicentrarchus labrax*, the system limited release during the gastric phase (~12.7% at 90 min) and enabled a substantially higher release during the intestinal phase (~82% at 360 min). These findings suggest preferential intestinal release under the applied in vitro conditions, rather than confirmed targeted delivery in vivo.

When incorporated into commercial aquafeed pellets, the encapsulated formulation delayed several storage‐related deterioration processes under accelerated aging conditions. Compared with the control and free EEO diets, encapsulated EEO was associated with lower moisture uptake and aw increase, reduced PV development, improved lipid and ω−3 retention, better preservation of antioxidant capacity and TPC, and lower losses in pellet durability, hardness, fines resistance, and water stability. These results suggest that the ulvan–lecithin microstructure may contribute to both chemical and physical stabilization of phytobiotic‐enriched aquafeeds. However, the individual contributions of antioxidant activity, moisture barrier effects, oxygen diffusion limitation, and matrix‐mediated protection were not quantified independently.

The findings support the potential of ulvan–lecithin microencapsulation as a proof‐of‐concept strategy for stabilizing volatile phytobiotics, modulating in vitro release behavior, and improving storage performance in functional aquafeeds. Nevertheless, the present work should not be interpreted as confirmation of biological efficacy or industrial feasibility. In vivo feeding trials are required to confirm bioavailability, feed intake, nutrient utilization, growth performance, immune response, gut health, and fish health outcomes. In addition, process‐scale validation, comparison with industrially scalable technologies such as spray drying, and techno‐economic assessment are necessary before commercial aquafeed implementation can be evaluated.

## Author Contributions

Conceptualization: Magdalini Krokida, Chrysanthos Stergiopoulos, Sofia Papadaki, and Nikoletta Tricha. Data curation: Nikoletta Tricha. Formal analysis: Nikoletta Tricha and Chrysanthos Stergiopoulos. Funding acquisition: Magdalini Krokida. Investigation: Nikoletta Tricha and Apostolos Tsoumanis. Methodology: Chrysanthos Stergiopoulos and Nikoletta Tricha. Project administration: Sofia Papadaki and Magdalini Krokida. Resources: Sofia Papadaki. Software: Chrysanthos Stergiopoulos and Nikoletta Tricha. Supervision: Sofia Papadaki and Magdalini Krokida. Validation: Chrysanthos Stergiopoulos and Nikoletta Tricha. Visualization: Nikoletta Tricha. Writing – original draft preparation: Nikoletta Tricha and Chrysanthos Stergiopoulos. Writing – review & editing: Chrysanthos Stergiopoulos and Nikoletta Tricha.

## Funding

This study was funded by the European Commission (Grant 101086261).

## Ethics Statement

This study did not involve live animals, animal experimentation, or human participants.

## Conflicts of Interest

The authors declare no conflicts of interest.

## Supporting Information

Additional supporting information can be found online in the Supporting Information section.

## Supporting information


**Supporting Information** Supporting File S1: Supporting tables including cumulative release profiles under PBS and simulated gastrointestinal conditions, storage stability data (moisture content, water activity, peroxide value, lipid retention, antioxidant capacity, total phenolic content), pellet quality parameters (PDI, hardness, fines content, and water stability), and corresponding two‐way ANOVA outputs supporting the results presented in the manuscript.

## Data Availability

The data that supports the findings of this study are available in the Supporting Information of this article.
