# Supplementary material for: Ulvan Microparticles for Encapsulation of Eucalyptus Essential Oil: Characterization, Release Behavior, and Stability in Aquafeeds
Source: Aquac Nutr. 2026 Aug 9;2026:3902818. doi: 10.1155/anu/3902818 (PMC13454364; doi:10.1155/anu/3902818)
Supplement: Supplementary file 1 [file ANU-2026-3902818-s001.docx]

**Table S1.** PBS Release Data

| **Time (h)** | **Cumulative Release (%)** | **SD Release (%)** |
| --- | --- | --- |
| 0 | 0 | 0 |
| 0.25 | 10.8 | 0.65 |
| 0.5 | 16.7 | 0.83 |
| 1 | 25.2 | 1.13 |
| 2 | 38.1 | 1.52 |
| 3 | 48.4 | 1.94 |
| 6 | 63.9 | 1.92 |
| 12 | 77.5 | 1.94 |
| 24 | 90.2 | 1.8 |

**Table S2.** Gastrointestinal Release Data

| **Time (min)** | **Phase** | **Cumulative Release (%)** | **SD (%)** |
| --- | --- | --- | --- |
| 0 | Gastric | 0 | 0 |
| 15 | Gastric | 4.3 | 0.43 |
| 30 | Gastric | 6.7 | 0.58 |
| 60 | Gastric | 10.1 | 0.64 |
| 90 | Gastric | 12.7 | 0.83 |
| 120 | Intestinal | 34.2 | 2.14 |
| 180 | Intestinal | 60.0 | 3.62 |
| 240 | Intestinal | 72.7 | 4.40 |
| 360 | Intestinal | 82.0 | 4.91 |

Time refers to total digestion time. The intestinal phase began after completion of the 90-min gastric phase; therefore, 120 min represents the first intestinal sampling point.

**Table S3.** Two-way ANOVA outputs for the effects of formulation, storage time, and their interaction on moisture content.

| **Parameter** | **Source of variation** | **SS** | **df** | **MS** | **F-value** | **p-value** | **Partial η²** |
| --- | --- | --- | --- | --- | --- | --- | --- |
| Moisture content | Formulation | 11.120 | 2 | 5.560 | 1637.981 | 4.49 × 10⁻³⁶ | 0.989 |
| Moisture content | Storage time | 38.072 | 5 | 7.614 | 2243.174 | 7.80 × 10⁻⁴⁴ | 0.997 |
| Moisture content | Formulation × storage time | 5.598 | 10 | 0.560 | 164.929 | 5.78 × 10⁻²⁷ | 0.979 |
| Moisture content | Error | 0.122 | 36 | 0.003 | — | — | — |
| Moisture content | Total | 54.912 | 53 | — | — | — | — |

Note: Partial η² was calculated as SS_effect_ / (SS_effect_ + SS_error_).

**Table S4.** Moisture content (% w/w) of samples during storage

| **Time (d)** | **Control** | **Free EEO** | **Encapsulated EEO** |
| --- | --- | --- | --- |
| 0 | 8.2 ± 0.25ᵃᴬ | 8.3 ± 0.22ᵃᴬ | 8.3 ± 0.28ᵃᴬ |
| 3 | 9.1 ± 0.32ᵇᴬ | 8.8 ± 0.29ᵃᵇᴬ | 8.6 ± 0.27ᵃᵇᴬ |
| 7 | 9.8 ± 0.30ᶜᴮ | 9.2 ± 0.26ᵇᶜᴬᴮ | 8.9 ± 0.25ᵇᶜᴬ |
| 10 | 10.4 ± 0.28ᶜᴮ | 9.6 ± 0.27ᶜᵈᴬ | 9.1 ± 0.26ᶜᵈᴬ |
| 14 | 11.2 ± 0.24ᵈᴮ | 10.0 ± 0.23ᵈᵉᴬ | 9.4 ± 0.29ᵈᵉᴬ |
| 28 | 12.0 ± 0.31ᵉ^C^ | 10.6 ± 0.34ᵉᴮ | 9.8 ± 0.30ᵉᴬ |

Values are expressed as mean ± standard deviation (n = 3). Different lowercase superscript letters within the same column indicate significant differences over storage time within the same formulation (p < 0.05). Different uppercase superscript letters within the same row indicate significant differences among formulations at the same storage time point (p < 0.05).

**Table S5.** Two-way ANOVA outputs for the effects of formulation, storage time, and their interaction on water activity (aw).

| **Parameter** | **Source of variation** | **SS** | **df** | **MS** | **F-value** | **p-value** | **Partial η²** |
| --- | --- | --- | --- | --- | --- | --- | --- |
| Water activity (aw) | Formulation | 0.01723 | 2 | 0.00861 | 3080.788 | 5.67 × 10⁻⁴¹ | 0.994 |
| Water activity (aw) | Storage time | 0.05946 | 5 | 0.01189 | 4252.664 | 8.03 × 10⁻⁴⁹ | 0.998 |
| Water activity (aw) | Formulation × storage time | 0.00873 | 10 | 0.00087 | 312.253 | 7.38 × 10⁻³² | 0.989 |
| Water activity (aw) | Error | 0.00010 | 36 | 0.000003 | — | — | — |
| Water activity (aw) | Total | 0.08552 | 53 | — | — | — | — |

Note: Partial η² was calculated as SS_effect_ / (SS_effect_ + SS_error_).

**Table S6.** Water activity (aw) of samples during storage time

| **Time (d)** | **Control** | **Free EEO** | **Encapsulated EEO** |
| --- | --- | --- | --- |
| 0 | 0.520 ± 0.004ᵃᴬ | 0.524 ± 0.003ᵃᴬ | 0.524 ± 0.003ᵃᴬ |
| 3 | 0.555 ± 0.006ᵇᴮ | 0.544 ± 0.005ᵇᴬᴮ | 0.536 ± 0.004ᵃᵇᴬ |
| 7 | 0.583 ± 0.007ᶜᴮ | 0.559 ± 0.005ᶜᴬ | 0.548 ± 0.004ᵇᶜᴬ |
| 10 | 0.607 ± 0.007ᵈ^C^ | 0.575 ± 0.006ᵈᴮ | 0.555 ± 0.005ᶜᵈᴬ |
| 14 | 0.638 ± 0.008ᵉ^C^ | 0.591 ± 0.006ᵉᴮ | 0.567 ± 0.005ᵈᴬ |
| 28 | 0.670 ± 0.008ᶠ^C^ | 0.615 ± 0.007ᶠᴮ | 0.583 ± 0.006ᵉᴬ |

Values are expressed as mean ± standard deviation (n = 3). Different lowercase superscript letters within the same column indicate significant differences over storage time within the same formulation (p < 0.05). Different uppercase superscript letters within the same row indicate significant differences among formulations at the same storage time point (p < 0.05).

**Table S7.** Peroxide value (PV) of samples during storage

| **Time (d)** | **Control** | **Free EEO** | **Encapsulated EEO** |
| --- | --- | --- | --- |
| 0 | 4.0 ± 0.2ᵃᴬ | 4.0 ± 0.2ᵃᴬ | 4.0 ± 0.2ᵃᴬ |
| 3 | 8.0 ± 0.4ᵇ^C^ | 7.0 ± 0.3ᵇᴮ | 6.0 ± 0.3ᵇᴬ |
| 7 | 14.0 ± 0.8ᶜ^C^ | 11.0 ± 0.6ᶜᴮ | 9.0 ± 0.5ᶜᴬ |
| 10 | 19.0 ± 1.0ᵈ^C^ | 15.0 ± 0.8ᵈᴮ | 11.0 ± 0.6ᵈᴬ |
| 14 | 25.0 ± 1.5ᵉ^C^ | 19.0 ± 1.1ᵉᴮ | 14.0 ± 0.9ᵉᴬ |
| 28 | 38.0 ± 2.2ᶠ^C^ | 28.0 ± 1.6ᶠᴮ | 20.0 ± 1.2ᶠᴬ |

Values are expressed as mean ± standard deviation (n = 3). Different lowercase superscript letters within the same column indicate significant differences over storage time within the same formulation (p < 0.05). Different uppercase superscript letters within the same row indicate significant differences among formulations at the same storage time point (p < 0.05).

**Table S8.** Two-way ANOVA outputs for the effects of formulation, storage time, and their interaction on peroxide value (PV).

| **Parameter** | **Source of variation** | **SS** | **df** | **MS** | **F-value** | **p-value** | **Partial η²** |
| --- | --- | --- | --- | --- | --- | --- | --- |
| Peroxide value (PV) | Formulation | 492.926 | 2 | 246.463 | 309.512 | 2.09 × 10⁻²³ | 0.945 |
| Peroxide value (PV) | Storage time | 3588.981 | 5 | 717.796 | 901.419 | 9.53 × 10⁻³⁷ | 0.992 |
| Peroxide value (PV) | Formulation × storage time | 319.296 | 10 | 31.930 | 40.098 | 1.62 × 10⁻¹⁶ | 0.918 |
| Peroxide value (PV) | Error | 28.667 | 36 | 0.796 | — | — | — |
| Peroxide value (PV) | Total | 4429.870 | 53 | — | — | — | — |

Note: Partial η² was calculated as SS_effect_ / (SS_effect_ + SS_error_).

**Table S9.** Lipid retention (%) of samples during storage

| **Time (d)** | **Control** | **Free EEO** | **Encapsulated EEO** |
| --- | --- | --- | --- |
| 0 | 100.00 ± 0.80ᵃᴬ | 100.00 ± 0.80ᵃᴬ | 100.00 ± 0.80ᵃᴬ |
| 3 | 96.35 ± 1.10ᵃ^b^ᴬ | 97.91 ± 1.00ᵃᵇᴬ | 98.44 ± 0.95ᵃ^b^ᴬ |
| 7 | 93.23 ± 1.30^bc^ᴬ | 95.81 ± 1.10ᵇᶜᴬ | 97.40 ± 1.00^ab^ᴬ |
| 10 | 90.63 ± 1.50^cd^ᴮ | 93.72 ± 1.20ᶜ^d^ᴬᴮ | 96.35 ± 1.05^bc^ᴬ |
| 14 | 89.06 ± 1.60^dB^ | 92.67 ± 1.25ᵈᴬ | 95.83 ± 1.10ᶜᴬ |
| 28 | 87.50 ± 1.80^dC^ | 92.15 ± 1.35^dB^ | 95.83 ± 1.15ᶜᴬ |

Values are expressed as mean ± standard deviation (n = 3). Different lowercase superscript letters within the same column indicate significant differences over storage time within the same formulation (p < 0.05). Different uppercase superscript letters within the same row indicate significant differences among formulations at the same storage time point (p < 0.05).

**Table S10.** Antioxidant capacity (mg TE/g dw) of samples during storage

| **Time (d)** | **Control** | **Free EEO** | **Encapsulated EEO** |
| --- | --- | --- | --- |
| 0 | 0.25 ± 0.02^aB^ | 0.48 ± 0.024^aA^ | 0.52 ± 0.019^aA^ |
| 3 | 0.23 ± 0.018^abB^ | 0.45 ± 0.020^abA^ | 0.50 ± 0.035^aA^ |
| 7 | 0.21 ± 0.015^abC^ | 0.42 ± 0.030^bcB^ | 0.48 ± 0.016^aA^ |
| 10 | 0.20 ± 0.012^abcC^ | 0.39 ± 0.019^cB^ | 0.46 ± 0.036^aA^ |
| 14 | 0.19 ± 0.010^bcC^ | 0.36 ± 0.016^dB^ | 0.46 ± 0.032^aA^ |
| 28 | 0.18 ± 0.010^cC^ | 0.34 ± 0.019^dB^ | 0.45 ± 0.018^aA^ |

Values are expressed as mean ± standard deviation (n = 3). Different lowercase superscript letters within the same column indicate significant differences over storage time within the same formulation (p < 0.05). Different uppercase superscript letters within the same row indicate significant differences among formulations at the same storage time point (p < 0.05).

**Table S11.** Changes in total phenolic content (TPC) during storage

| **Time (d)** | **Control** | **Free EEO** | **Encapsulated EEO** |
| --- | --- | --- | --- |
| 0 | 0.32 ± 0.027ᵃᴮ | 0.76 ± 0.036ᵃᴬ | 0.82 ± 0.037ᵃᴬ |
| 3 | 0.30 ± 0.026ᵃ^C^ | 0.70 ± 0.025ᵃᵇᴮ | 0.78 ± 0.036ᵃᴬ |
| 7 | 0.28 ± 0.030ᵃᵇ^C^ | 0.64 ± 0.047ᵇᶜᴮ | 0.75 ± 0.050ᵃᴬ |
| 10 | 0.25 ± 0.035ᵇᶜ^C^ | 0.58 ± 0.035ᶜᵈᴮ | 0.72 ± 0.045ᵃᵇᴬ |
| 14 | 0.23 ± 0.032ᶜ^C^ | 0.54 ± 0.025ᵈᴮ | 0.70 ± 0.052ᵇᴬ |
| 28 | 0.21 ± 0.025ᶜ^C^ | 0.51 ± 0.017ᵈᴮ | 0.69 ± 0.037ᵇᴬ |

Values are expressed as mean ± standard deviation (n = 3). Different lowercase superscript letters within the same column indicate significant differences over storage time within the same formulation (p < 0.05). Different uppercase superscript letters within the same row indicate significant differences among formulations at the same storage time point (p < 0.05).

**Table S12.** Two-way ANOVA outputs for the effects of formulation, storage time, and their interaction on pellet durability index (PDI).

| **Parameter** | **Source of variation** | **SS** | **df** | **MS** | **F-value** | **p-value** | **Partial η²** |
| --- | --- | --- | --- | --- | --- | --- | --- |
| PDI | Formulation | 12.029 | 2 | 6.015 | 26.818 | 7.39 × 10⁻⁸ | 0.598 |
| PDI | Storage time | 160.603 | 5 | 32.121 | 143.221 | 1.03 × 10⁻²² | 0.952 |
| PDI | Formulation × storage time | 24.254 | 10 | 2.425 | 10.815 | 3.56 × 10⁻⁸ | 0.750 |
| PDI | Error | 8.074 | 36 | 0.224 | — | — | — |
| PDI | Total | 204.960 | 53 | — | — | — | — |

Note: Partial η² was calculated as SS_effect_ / (SS_effect_ + SS_error_).

**Table S13.** Pellet Durability Index (PDI, %) during storage

| **Time (d)** | **Control** | **Free EEO** | **Encapsulated EEO** |
| --- | --- | --- | --- |
| 0 | 96.10 ± 0.13ᵃᴬ | 95.81 ± 0.46ᵃᴬ | 95.59 ± 0.34ᵃᴬ |
| 3 | 95.57 ± 0.35ᵃᴬ | 95.27 ± 0.28ᵃᴬ | 95.70 ± 0.11ᵃᴬ |
| 7 | 94.80 ± 0.43ᵇᴬ | 94.58 ± 0.27ᵃᵇᴬ | 94.85 ± 0.31ᵃᵇᴬ |
| 10 | 93.74 ± 0.28ᶜᴮ | 94.23 ± 0.24ᵇᴬᴮ | 94.77 ± 0.45ᵃᵇᴬ |
| 14 | 92.14 ± 0.66ᵈᴮ | 93.86 ± 0.70ᵇᴬ | 94.15 ± 0.67ᵇᴬ |
| 28 | 88.46 ± 0.53ᵉ^C^ | 90.81 ± 0.89ᶜᴮ | 92.68 ± 0.55ᶜᴬ |

Values are expressed as mean ± standard deviation (n = 3). Different lowercase superscript letters within the same column indicate significant differences over storage time within the same formulation (p < 0.05). Different uppercase superscript letters within the same row indicate significant differences among formulations at the same storage time point (p < 0.05).

**Table S14.** Hardness (N) of samples during storage

| **Time (d)** | **Control** | **Free EEO** | **Encapsulated EEO** |
| --- | --- | --- | --- |
| 0 | 55.37 ± 1.05ᵃᴬ | 54.34 ± 2.10ᵃᴬ | 54.82 ± 0.84ᵃᴬ |
| 3 | 53.88 ± 0.78ᵃᵇᴬ | 53.52 ± 0.69ᵃᵇᴬ | 52.64 ± 0.78ᵃᵇᴬ |
| 7 | 49.34 ± 0.71ᵇᶜᴬ | 49.70 ± 1.68ᵇᶜᴬ | 51.98 ± 0.24ᵃᵇᴬ |
| 10 | 47.83 ± 2.27ᶜᵈᴬ | 49.40 ± 1.71ᵇᶜᴬ | 49.07 ± 1.11ᵇᶜᴬ |
| 14 | 43.70 ± 1.08ᵈᴮ | 45.39 ± 1.83ᶜᴬᴮ | 47.39 ± 0.66ᶜᴬ |
| 28 | 25.25 ± 3.28ᵉᴮ | 34.21 ± 1.65ᵈᴬᴮ | 43.37 ± 2.72ᵈᴬ |

Values are expressed as mean ± standard deviation (n = 3). Different lowercase superscript letters within the same column indicate significant differences over storage time within the same formulation (p < 0.05). Different uppercase superscript letters within the same row indicate significant differences among formulations at the same storage time point (p < 0.05).

**Table S15.** Fines content (%) during storage

| **Time (d)** | **Control** | **Free EEO** | **Encapsulated EEO** |
| --- | --- | --- | --- |
| 0 | 1.38 ± 0.23ᵃᴬ | 1.50 ± 0.11ᵃᴬ | 1.59 ± 0.21ᵃᴬ |
| 3 | 2.02 ± 0.34ᵃᵇᴬ | 1.74 ± 0.19ᵃᴬ | 1.81 ± 0.24ᵃᴬ |
| 7 | 2.57 ± 0.07ᵃᵇᴬ | 2.32 ± 0.25ᵃᵇᴬ | 2.26 ± 0.17ᵃᵇᴬ |
| 10 | 3.33 ± 0.41ᵇᶜᴬ | 3.00 ± 0.41ᵇᶜᴬ | 2.59 ± 0.19ᵃᵇᴬ |
| 14 | 4.47 ± 0.47ᶜᴬ | 3.63 ± 0.41ᶜᴬ | 3.24 ± 0.61ᵇᴬ |
| 28 | 9.18 ± 1.08ᵈᴮ | 6.48 ± 0.88ᵈᴬ | 4.99 ± 0.59ᶜᴬ |

Values are expressed as mean ± standard deviation (n = 3). Different lowercase superscript letters within the same column indicate significant differences over storage time within the same formulation (p < 0.05). Different uppercase superscript letters within the same row indicate significant differences among formulations at the same storage time point (p < 0.05).

**Table S16.** Water stability (% retention) of samples during storage

| **Time (d)** | **Control** | **Free EEO** | **Encapsulated EEO** |
| --- | --- | --- | --- |
| 0 | 95.17 ± 0.43ᵃᴬ | 94.73 ± 0.36ᵃᴬ | 94.80 ± 0.30ᵃᴬ |
| 3 | 94.78 ± 0.37ᵃᴬ | 94.44 ± 0.24ᵃᴬ | 94.89 ± 0.13ᵃᴬ |
| 7 | 92.52 ± 0.20ᵇᴬ | 93.17 ± 0.40ᵇᴬ | 93.39 ± 0.58ᵇᴬ |
| 10 | 91.58 ± 0.40ᵇᴮ | 92.17 ± 0.82ᵇᴮ | 94.31 ± 0.70ᵃᵇᴬ |
| 14 | 90.30 ± 0.33ᶜᴮ | 91.02 ± 0.64ᶜᴮ | 93.14 ± 0.23ᵇᴬ |
| 28 | 84.66 ± 0.75ᵈ^C^ | 86.66 ± 0.78ᵈᴮ | 90.04 ± 0.69ᶜᴬ |

Values are expressed as mean ± standard deviation (n = 3). Different lowercase superscript letters within the same column indicate significant differences over storage time within the same formulation (p < 0.05). Different uppercase superscript letters within the same row indicate significant differences among formulations at the same storage time point (p < 0.05).
